# A palladium complex of 2,2′-(propane-1,3-diylbis(oxy))dibenzenaminium chloride on SBA-15 as a returnable, environmental and selective nanostructured-catalyst in the Suzuki C–C coupling reaction

**DOI:** 10.1039/d5ra08145a

**Published:** 2026-04-27

**Authors:** Shirin Mohammadi, Mohsen Nikoorazm, Bahman Tahmasbi, Yunes Abbasi Tyula

**Affiliations:** a Department of Chemistry, Faculty of Science, Ilam University P. O. Box 69315516 Ilam Iran m.nikorazm@ilam.ac.ir b.tahmasbi@ilam.ac.ir

## Abstract

In this work, mesoporous SBA-15 was synthesized by a simple procedure using P123 and TEOS in HCl acidic conditions, followed by calcination at 550 °C. 2,2′-(Propane-1,3-diylbis(oxy))dibenzenaminium chloride ligand (PO(BA)) as ligand was synthesized from 2-nitrophenol and 1,3-dibromopropane, following reduction of nitro groups. The step-by-step synthesis of the PO(BA) ligand was confirmed by ^1^H NMR and ^13^C NMR. 3-Iodopropyltriethoxysilane (IPTES) was synthesized from 3-chloropropyltriethoxysilane (CPTES) in dry acetone. Then, the mesoporous SBA-15 surface was modified by IPTES and next it was functionalized by PO(BA). Finally, immobilized PO(BA) on SBA-15 became complex with palladium acetate (Pd-PO(BA)@SBA-15). The prepared Pd-PO(BA)@SBA-15 was characterized by SEM, ICP, TGA/DSC, EDS, BET/BJH, and WDX techniques. TGA and BET/BJH methods showed high thermal stability of this catalyst up to 230 °C and a high surface area for this catalyst. Then, the catalytic usage of Pd-PO(BA)@SBA-15 was investigated in the selective carbon–carbon bond formation. Various aryl halides (including aryl iodides (Ar–I) and aryl bromides (Ar–Br), having electron-donating or electron-withdrawing functional groups) and some derivatives of phenylboronic acid (such as phenylboronic acid, 4-methoxyphenylboronic acid and 4-formylphenylboronic acid) were investigated and all biphenyl products were obtained with high yields and TOF values. NMR spectroscopy was used to determine the synthesized biphenyl products. Pd-PO(BA)@SBA-15 catalyst was shown to be reusable without significant loss in its performance in the Suzuki–Miyaura cross-coupling reaction.

## Abstract 2

Modified SBA-15 was functionalized by a new complex of palladium, and was characterized by SEM, ICP, TGA/DSC, EDS, BET/BJH, and WDX techniques. Then, its catalytic activity was investigated in the selective C–C coupling reaction.

## Graphical abstract

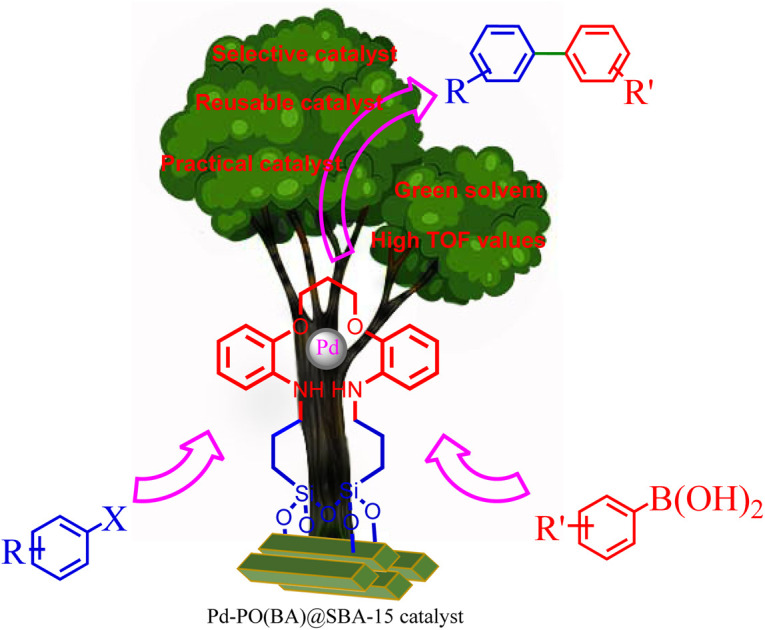

## Introduction

The C–C bond formation, which plays a usual and great role in the synthesis of organic materials to forming preparation of hydrocarbons, UV screens, conductive polymers, herbicides, pharmaceuticals, natural products, polymerization processes, ligands, advanced materials, insecticides, agrochemicals, *etc.*^1^ Homologous of C–C coupling reactions (include coupling of two similar reactants to synthesis of a symmetric molecule) and cross-coupling of C–C reactions (include coupling of two quite different reactants to form a new molecule) are two general categories for C–C bond formation.^2^ The Suzuki reaction is an important catalytic C–C coupling reaction, including the coupling of an arylboronic acid with an aryl halide,^2,3^ which can produce various drugs, *e.g.*, valsartan, lapatinib, losartan, atazanavir, diflunisal, and clonazepam.^2^ The Suzuki reaction is usually performed in the presence of homogeneous and heterogeneous Pd-catalyts.^3*b*,4^ Given the sensitive ligands and high price of Pd-catalysts, heterogeneous-based palladium catalysts are suitable over palladium-containing homogeneous catalysts due to the recoverability and reusability of heterogeneous catalysts.^3*b*,5^ Therefore, the heterogeneous Pd-catalysts, especially immobilized palladium on the surface of various supports, have attracted much attention in the field of catalysis.^3*b*^ In this regard, biochar, CMK-3, graphene oxide(GO), polymers, mesoporous/amorphous silica materials, boehmite nanoparticles, MOFs (metal–organic frameworks), magnetic nanoparticles, zeolites, carbon nanomaterials, *etc.* Were used as support for immobilization of practical catalysts.^6^ Among mesoporous silica materials, SBA-15 is an ideal nanostructure for immobilization of catalyst active species, which has excellent stability, great specific surface area, including modifiable silanol groups, and customizable pore size.^7^ SBA-15 is classified as a mesoporous material in the IUPAC classification (type IV isotherm with H1 hysteresis).^8^ Therefore, supported catalysts on SBA-15 have high catalytic activity (like homogeneous catalysts), and also these catalysts are recyclable (like heterogeneous catalysts). Therefore, in this work, SBA-15 was synthesized and then modified by IPTES, and then functionalized with PO(BA) for immobilization of a palladium complex (Pd-PO(BA)@SBA-15) as an efficient, returnable and practical nanocatalyst in the Suzuki C–C cross-coupling reaction.

## Experimental

### Synthesis of SBA-15

As the first step in the catalyst synthesis, mesoporous SBA-15 was prepared as a catalyst support. The synthesis process of SBA-15 was carried out according to the previous report. This process includes: pluronic 123 (4 g) was dissolved in 130 ml of deionized water and 20 ml HCl 37% and stirred at 40 °C for 90 min. Then, tetraethylorthosilicate (8.5 g) was added dropwise and stirred at room temperature until it became a clear solution. Next, the solution was stirred at 40 °C for 24 h. Then, it was kept in an autoclave at 100 °C for 48 h. The white precipitate obtained was recovered by filtration, washed with H_2_O and dried at room temperature. Finally, the template was dried and calcined at 550 °C with 2 °C min^−1^ for 5 h. The obtained white powder is SBA-15.

### Synthesis of 3-iodopropyltriethoxysilane (IPTES)

**Scheme 1:**
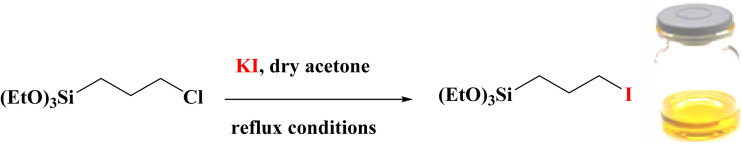
Synthesis of 3-iodopropyltrimethoxysilane (IPTES).

KI (5 mmol) was added in dry acetone (25 ml), and then 3-chloropropyltriethoxysilane (5 mmol) was injected dropwise under stirring. The mixture was mixed under N_2_ atmosphere at 50 °C for 24 h (Scheme 1). Finally, the mixture was filtered, and then, the acetone solvent was removed by evaporation. The remaining liquid is 3-iodopropyltriethoxysilane (IPTES).

### Synthesis of 2,2′-(propane-1,3-diylbis(oxy))dibenzenaminium chloride (PO(BA))

**Scheme 2:**
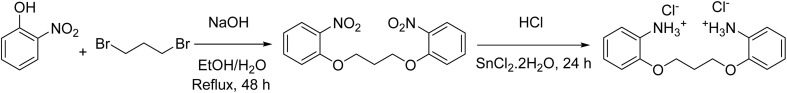
Synthesis of 2,2′-(propane-1,3-diylbis(oxy))dibenzenaminium chloride (PO(BA)).

First, 5.65 g (40 mmol) of 2-nitrophenol was completely dissolved in a flask containing 35 ml of ethanol. Then, 1.6 g (40 mmol) of NaOH was dissolved in 10 ml of water and added to the above solution. In this case, the color of the resulting solution changed from light yellow to red. In the next step, 4.04 g (20 mmol) of 1,3-dibromopropane was dissolved in 20 ml of ethanol and added dropwise to the above solution. Then, the solution was refluxed for 48 h. After about 24 h from the start of reflux, a yellow precipitate began to form on the wall of the container. At the end of the reflux time, a light yellow precipitate had formed in the flask, which was filtered and washed with ethanol and recrystallized in ethanol solvent for further purification (Precipitate weight 5.33 g, yield 41.75% after recrystallization and melting point 110 °C). At this stage, 1,3-bis(2-nitrophenoxy)propane was synthesized, which was identified and confirmed by ^1^H NMR and ^13^C NMR (Scheme 2). ^1^H NMR (250 MHz, *δ* ppm DMSO-*d*_6_): 7.84 (d, *J* = 7.5 Hz, 2H), 7.62 (t, *J* = 7.5 Hz, 2H), 7.34 (d, *J* = 7.5 Hz, 2H), 7.08 (t, *J* = 7.5 Hz, 2H), 4.31 (t, *J* = 7.5 Hz, 4H), 2.20 (quin, *J* = 7.5 Hz, 2H) ppm. ^13^C NMR (100 MHz, DMSO-*d*_6_): 151.6, 139.9, 134.9, 125.4, 121.0, 115.4, 65.9, 28.5 ppm.

To synthesize PO(BA), the method reported by Cannon and colleagues^9^ and optimization of their method for the reduction of the nitro group were used. In a 250 ml flask, 2.06 g of 1,3-bis(2-nitrophenoxy)propane was dissolved in 100 ml of hydrochloric acid (5 N) at a temperature of 40–50 °C using a magnetic stirrer. Then, 10 g of SnCl_2_·2H_2_O was added gradually to the above solution and the solution was refluxed. After 24 hours, the color of the solution changed from light yellow to white. The reaction was stopped at this stage and the reaction mixture was filtered, and the amine salt was obtained as a white precipitate (Scheme 2). The synthesized 2,2′-(propane-1,3-diylbis(oxy))dibenzenaminium chloride (PO(BA)) was identified and confirmed by ^1^H NMR and ^13^C NMR. ^1^H NMR (250 MHz, *δ* ppm DMSO-*d*_6_): 7.42–7.38 (m, 2H), 7.36–7.32 (m, 2H), 7.22 (d, *J* = 7.5 Hz, 2H), 7.00 (t, *J* = 7.5 Hz, 2H), 4.33 (t, *J* = 5 Hz, 4H), 3.80 (br, 6H), 2.23 (s, 2H) ppm. ^13^C NMR (100 MHz, DMSO-*d*_6_): 151.8, 129.7, 124.2, 123.4, 121.0, 113.6, 65.5, 28.7 ppm.

### Synthesis of Pd-PO(BA)@SBA-15 catalyst

**Scheme 3:**
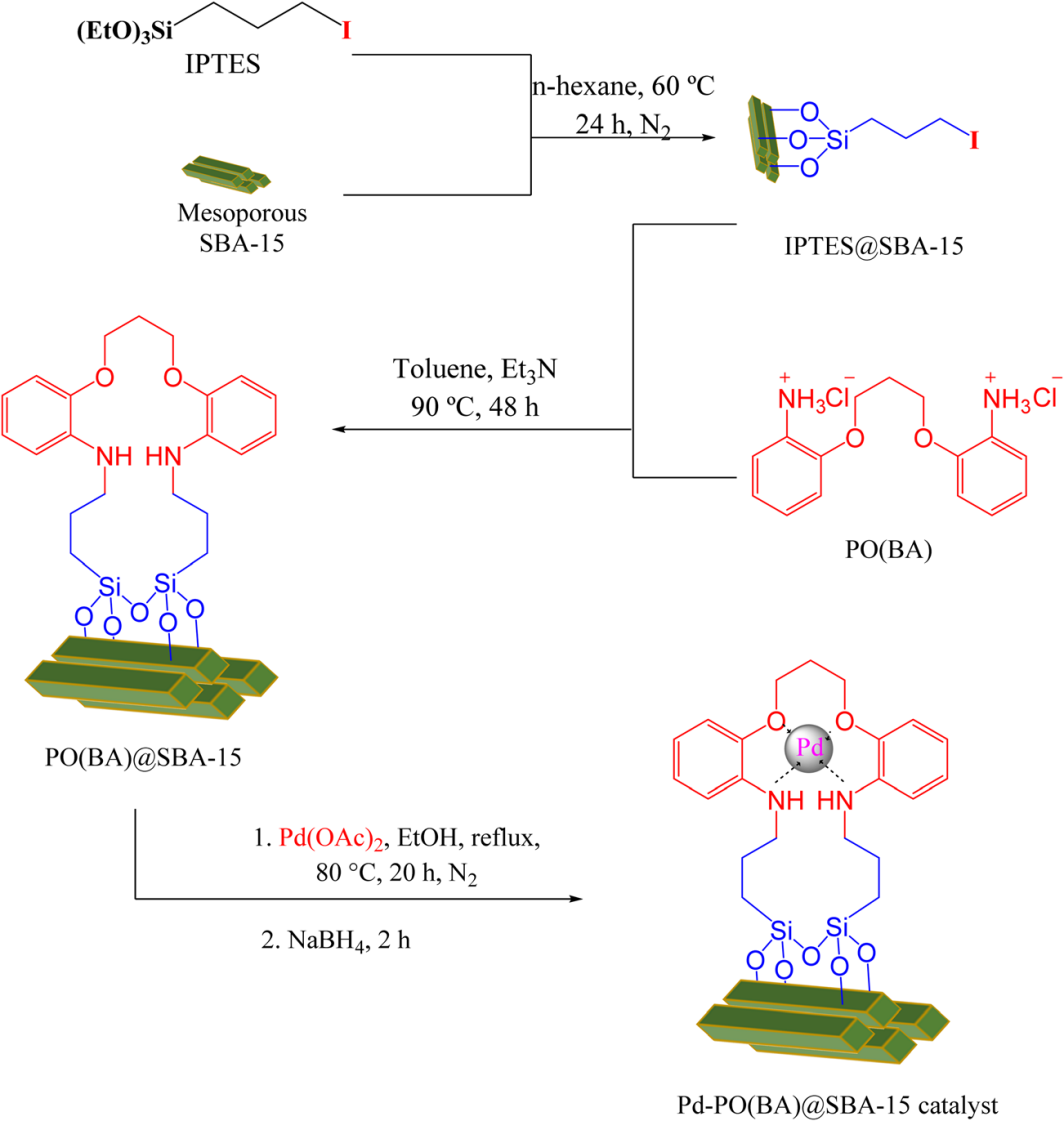
Synthesis of Pd-PO(BA)@SBA-15 preparation.

Mesoporous SBA-15 (1 g) was added to *n*-hexane (25 ml), and 1.5 ml of IPTES was injected, and further it was stirred at 60 °C for 24 h under N_2_ atmosphere. The modified SBA-15 (IPTES@SBA-15) was isolated with simple filtration. Then, IPTES@SBA-15 (1.2 g) and 1.5 mmol OF PO(BA) ligand were dispersed in toluene (30 ml). Then, 9 mmol of triethylamine (Et_3_N) was injected dropwise under stirring. The mixture was stirred for 48 h at 90 °C under reflux conditions. The PO(BA)@SBA-15 (above functionalized SBA-15) was isolated with simple filtration, washed with DMSO and further with EtOH. At the end step, PO(BA)@SBA-15 (0.5 g) was mixed with palladium acetate (0.25 mmol) in ethanol for 20 h under reflux conditions and N_2_ atmosphere. After that, 0.3 mmol (0.018 g) of NaBH_4_ was added, and stirring was continued for 2 h. The immobilized Pd-PO(BA) on the surface of SBA-15 (Pd-PO(BA)@SBA-15 catalyst) was isolated by filtration and washing with H_2_O and further ethanol (Scheme 3).

### Procedure for the synthesizing of biphenyl catalyzed by Pd-PO(BA)@SBA-15

**Scheme 4:**
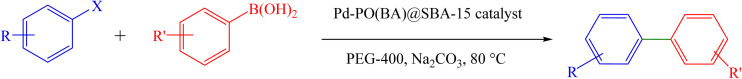
Synthesis of biphenyls in the presence of Pd-PO(BA)@SBA-15 catalyst.

A mixture of arylboronic acid (1 mmol), aryl halide (1 mmol), Na_2_CO_3_ (0.31 g, 3 mmol), and Pd-PO(BA)@SBA-15 catalyst (12 mg) was mixed by a magnetic stirrer at 80 °C in PEG-400 (Scheme 4). The reaction progress was monitored by TLC analysis. After completion, the Pd-PO(BA)@SBA-15 catalyst was removed by filtration after the addition of water and ethyl acetate (EtOAc), and the remaining mixture after filtration was moved to a separator funnel containing water. Then the biphenyls were extracted in EtOAc.

### NMR spectral data

#### 4-Nitro-1,1′-biphenyl

^1^H NMR (300 MHz, *δ* ppm DMSO-*d*_6_): 8.29 (d, *J* = 9 Hz, 2H), 7.95 (d, *J* = 9 Hz, 2H), 7.77 (d, *J* = 6 Hz, 2H), 7.56–7.46 (m, 3H) ppm.

^13^C NMR (100 MHz, CDCl_3_): 147.6, 147.1, 138.8, 129.2, 128.9, 127.8, 127.4, 124.1 ppm.

#### 4-Methoxy-1,1′-biphenyl

^1^H NMR (300 MHz, *δ* ppm DMSO-*d*_6_): 7.59 (d, *J* = 6 Hz, 4H), 7.41 (t, *J* = 7.5 Hz, 2H), 7.29 (t, *J* = 7.5 Hz, 1H), 7.01 (d, *J* = 9 Hz, 2H), 3.78 (s, 3H) ppm.

^13^C NMR (100 MHz, CDCl_3_): 159.1, 140.8, 133.8, 128.7, 128.2, 126.8, 126.7, 114.2, 55.4 ppm.

#### [1,1′-Biphenyl]-4-carbaldehyde

^1^H NMR (300 MHz, *δ* ppm DMSO-*d*_6_): 10.04 (s, 1H), 7.98 (d, *J* = 9 Hz, 2H), 7.88 (t, *J* = 7.5 Hz, 2H), 7.75 (d, *J* = 6 Hz, 2H), 7.53–7.40 (m, 3H) ppm.

#### 4-Methoxy-4′-nitro-1,1′-biphenyl

^1^H NMR (300 MHz, *δ* ppm DMSO-*d*_6_): 8.25 (d, *J* = 9 Hz, 2H), 7.90 (d, *J* = 9 Hz, 2H), 7.75 (d, *J* = 9 Hz, 2H), 7.07 (d, *J* = 9 Hz, 2H), 3.81 (s, 3H) ppm.

^13^C NMR (100 MHz, CDCl_3_): 160.4, 147.2, 146.5, 131.0, 128.6, 127.1, 124.1, 114.6, 55.4 ppm.

#### 1,1′-Biphenyl

^1^H NMR (300 MHz, *δ* ppm CDCl_3_): 7.63 (d, *J* = 9 Hz, 4H), 7.48 (t, *J* = 6 Hz, 4H), 7.39 (d, *J* = 9 Hz, 2H) ppm.

^13^C NMR (100 MHz, CDCl_3_): 141.3, 128.8, 127.3, 127.2 ppm.

#### 4′-Nitro-[1,1′-biphenyl]-4-carbaldehyde

^1^H NMR (300 MHz, *δ* ppm CDCl_3_): 10.09 (s, 1H), 8.34 (d, *J* = 9 Hz, 2H), 8.02 (d, *J* = 9 Hz, 2H), 7.80 (d, *J* = 9 Hz, 4H) ppm.

^13^C NMR (100 MHz, CDCl_3_): 191.7, 147.7, 146.0, 144.5, 136.2, 130.5, 128.2, 128.1, 124.3 ppm.

#### 4-Chloro-1,1′-biphenyl

^1^H NMR (300 MHz, *δ* ppm CDCl_3_): 7.58–7.52 (m, 4H), 7.46 (t, *J* = 9 Hz, 4H), 7.40 (t, *J* = 3 Hz, 1H) ppm.

^13^C NMR (100 MHz, CDCl_3_): 140.0, 139.7, 133.4, 128.9, 128.9, 128.4, 127.6, 127.0 ppm.

#### 4-Methyl-1,1′-biphenyl

^1^H NMR (300 MHz, *δ* ppm CDCl_3_): 7.62 (d, *J* = 9 Hz, 2H), 7.53 (d, *J* = 6 Hz, 2H), 7.46 (t, *J* = 6 Hz, 2H), 7.35 (t, *J* = 6 Hz, 1H), 7.28 (t, *J* = 9 Hz, 2H), 2.43 (s, 3H) ppm.

^13^C NMR (100 MHz, CDCl_3_): 141.2, 138.4, 137.0, 129.5, 128.7, 127.0, 127.0, 21.1 ppm.

#### [1,1′-Biphenyl]-4-carbonitrile

^1^H NMR (300 MHz, *δ* ppm CDCl_3_): 7.73 (d, *J* = 9 Hz, 2H), 7.68 (d, *J* = 9 Hz, 2H), 7.60 (d, *J* = 9 Hz, 2H), 7.52–7.43 (m, 3H) ppm.

^13^C NMR (100 MHz, CDCl_3_): 145.7, 139.1, 132.6, 129.1, 128.7, 127.7, 127.2, 119.0, 110.9 ppm.

## Results and discussion

### Characterization of Pd-PO(BA)@SBA-15

#### SEM images

**Fig. 1:**
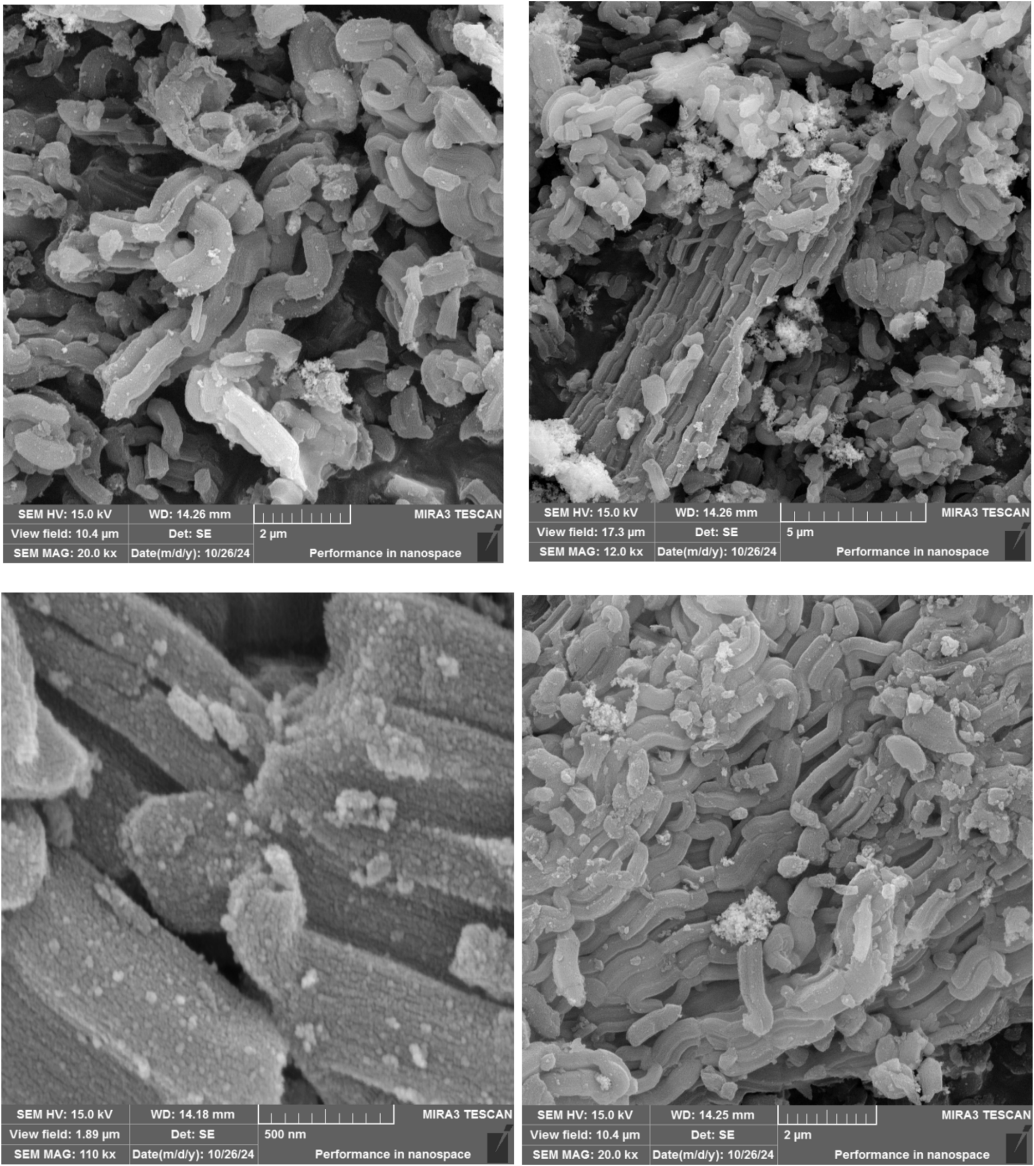
SEM images of Pd-PO(BA)@SBA-15.

A SEM instrument was used to study the various properties of Pd-PO(BA)@SBA-15 *e.g.* shape, morphology, and size of its particles (Fig. 1). The two SEM images in different scales (200 nm and 500 nm) of Pd-PO(BA)@SBA-15 catalyst are presented in Fig. 1.

#### Investigation of the elemental composition of Pd-PO(BA)@SBA-15

**Fig. 2:**
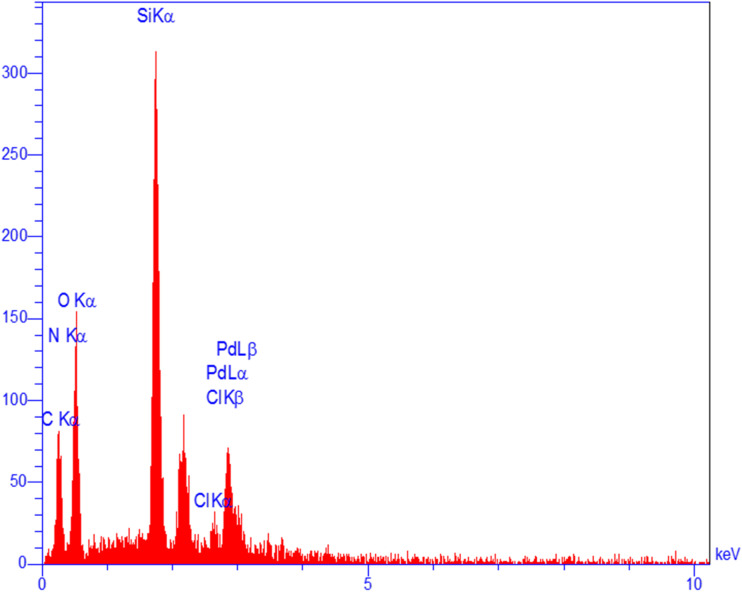
EDS diagram of Pd-PO(BA)@SBA-15.

In order to determine the elemental composition of Pd-PO(BA)@SBA-15, its EDS analysis was performed (Fig. 2), which indicates that this catalyst is formed from Si, O, Pd, C, N, and Cl species. Based on EDS results, N and C elements exist in the structure of Pd-PO(BA)@SBA-15, which indicates the surface modification of SBA-15 and its stabilization with PO(BA) ligand. Also, EDS analysis showed that this catalyst has palladium element, which confirmed well immobilization of Pd-complex on the surface of SBA-15.

**Fig. 3:**
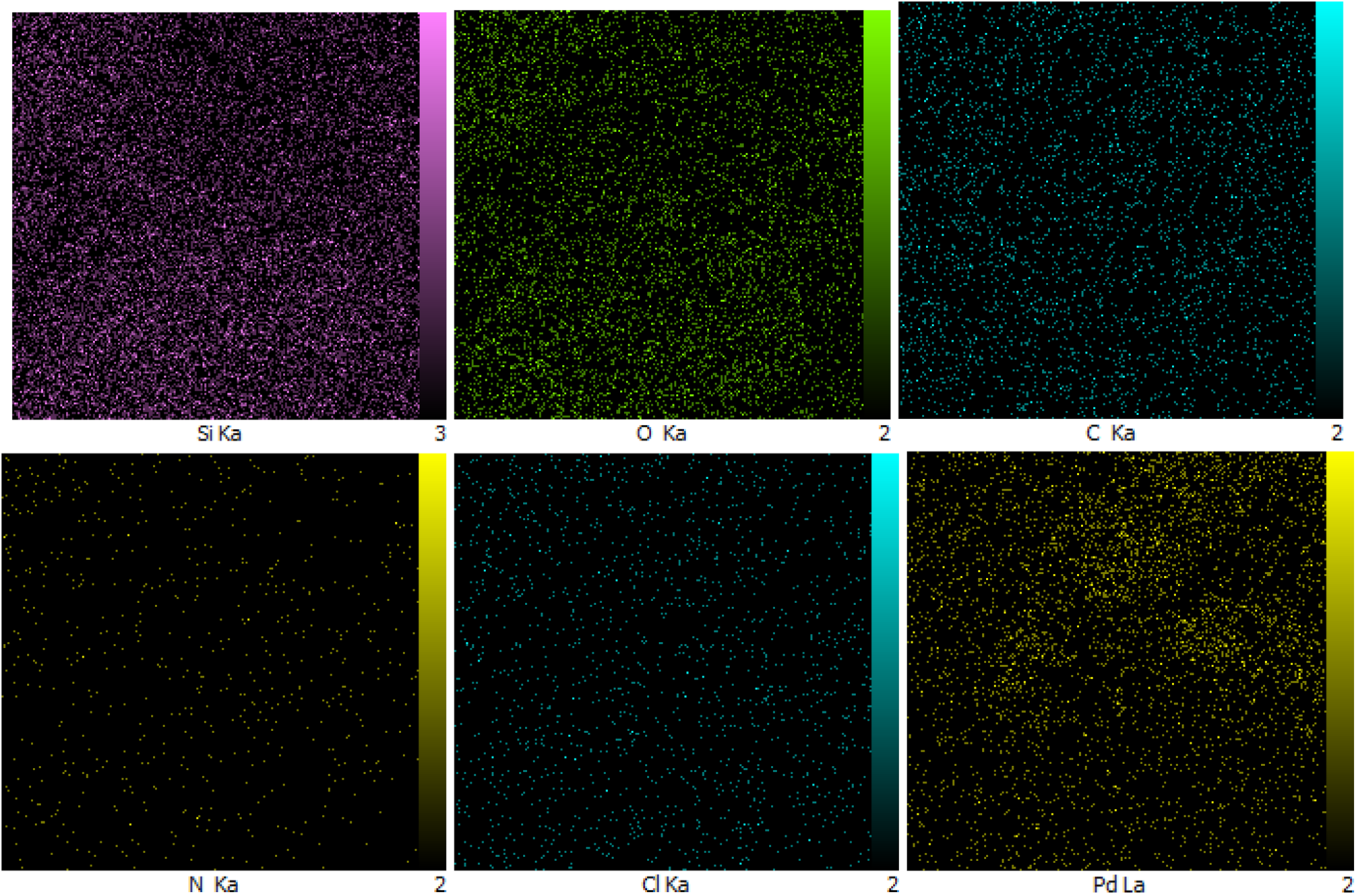
Elemental mapping for Pd-PO(BA)@SBA-15.

The distribution of all elements was studied by WDX analysis (Fig. 3). WDX analysis of Pd-PO(BA)@SBA-15 indicates a homogeneous distribution of Si, O, Pd, C, N, and Cl species.

Considering that palladium specie has the main active catalytic species in Pd-PO(BA)@SBA-15, the exact loaded palladium on the surface of modified SBA-15 was calculated by the ICP analysis, which was found to be 0.358 mmol g^−1^.

#### N_2_ adsorption–desorption isotherms study

**Fig. 4:**
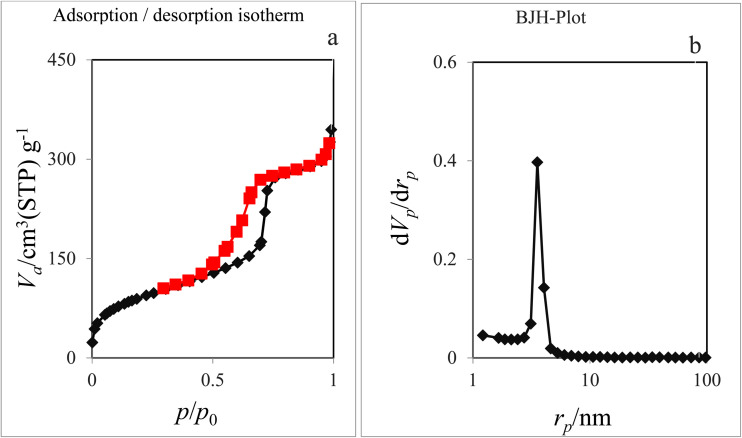
N_2_ adsorption–desorption isotherms (a) and BJH-Plot (b) of Pd-PO(BA)@SBA-15.

The nitrogen adsorption/desorption analysis was used to characterise structural and textural properties of Pd-PO(BA)@SBA-15. The obtained isotherms, BJH, and BET diagrams are illustrated in Fig. 4, and all output data are listed in Table 1. As displayed, SBA-15 and Pd-PO(BA)@SBA-15 surface areas are 754.41 m^2^ g^−1^ and 321.56 m^2^ g^−1^, respectively. Also, SBA-15 and Pd-PO(BA)@SBA-15 pore volume are 1.24 cm^3^ g^−1^ and 0.53 cm^3^ g^−1^, respectively. Also, SBA-15 and Pd-PO(BA)@SBA-15 pore diameters are 8.653 nm and 6.606 nm, respectively. These decreases in the textural and structural properties – *e.g.* surface area, pore volume, and pore diameters – of Pd-PO(BA)@SBA-15 than SBA-15 confirm the successful functionalization of SBA-15 and immobilization of Pd-complex on SBA-15′s channels.

**Table 1:** Textural and structural parameters of KIT-6 and Pd-PO(BA)@SBA-15

Sample	*S*_a_, _BET_ (m^2^ g^−1^)	Mean pore diameter (nm)	Total pore volume (cm^3^)
SBA-15	754.41 (ref. 10)	8.653 (ref. 10)	1.24 (ref. 10)
Pd-PO(BA)@SBA-15	321.56	6.606	0.5311

#### TGA study

**Fig. 5:**
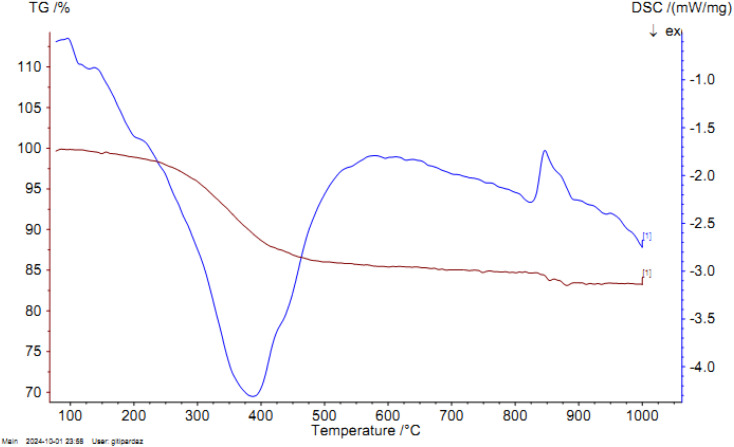
TGA diagram of Pd-PO(BA)@SBA-15 catalyst and DSC diagram of Pd-PO(BA)@SBA-15 catalyst.

The organic content and thermal stability of Pd-PO(BA)@SBA-15 were investigated by thermogravimetric analysis (TGA), as shown in Fig. 5. Also, the DSC diagram of Pd-PO(BA)@SBA-15 nanocatalyst is shown in Fig. 5. In the TGA curve of Pd-PO(BA)@SBA-15 catalyst, the weight loss before 230 °C (about 2%) is due to the evaporation of the solvents. Except for the solvent evaporation, no reduction in weight was observed up to 230 °C. Therefore, Pd-PO(BA)@SBA-15 catalyst is thermally stable up to 230 °C. Also, a reduction in weight occurs at 230 to 500 °C (about 17.50%), which is related to the organic layers on SBA-15, indicating functionalization of SBA-15 with organic layers and Pd-complex.

#### Catalytic studies of Pd-PO(BA)@SBA-15

After the synthesis and characterisation of Pd-PO(BA)@SBA-15 catalyst, its catalytic performance was investigated in the Suzuki C–C coupling reaction.

The synthesizing conditions of biphenyls through Suzuki C–C coupling reaction in the presence of Pd-PO(BA)@SBA-15 were optimized in the coupling of phenylboronic acid and iodobenzene (C_6_H_5_–I) under different conditions, including the amount of Pd-PO(BA)@SBA-15 catalyst (Table 2, entries 1–3), solvent type (Table 2, entries 3–7), reaction temperature (Table 2, entries 10 and 11), and base type (Table 2, entries 7–10). The obtained experimental data are listed in Table 2, indicating 12 mg of Pd-PO(BA)@SBA-15 catalyst using Na_2_CO_3_ (sodium carbonate) as a base in PEG-400 as a solvent at 80 °C for optimal reaction conditions (Table 2, entry 3). As outlined in Table 2, 12 mg of Pd-PO(BA)@SBA-15 catalyst was selected for the coupling of phenylboronic acid with aryl halides, because lower amounts of Pd-PO(BA)@SBA-15 catalyst is not enough for completion of C–C bond formation (Table 2, entries 1 and 2) and the amount catalyst increasing of Pd-PO(BA)@SBA-15 not significant affect in results of the C–C coupling reaction in the presence of this catalyst. Among various inorganic and organic solvents such as PEG-400 (polyethylene glycol 400), EtOH, DMF (dimethylformamide), DMSO (dimethyl sulfoxide), and H_2_O, PEG-400 as a suitable solvent provided the best environmental conditions for the coupling of phenylboronic acid with C_6_H_5_–I in the presence of Pd-PO(BA)@SBA-15 catalyst (Table 2, entries 3–7). So, PEG-400 was selected as a green solvent over many traditional organic solvents due to its interesting advantages, *e.g.* non-toxicity, bio-degradability, non-flammability, readily recyclability, thermal stability, abundant availability, and cost-effectiveness.^11^ Among various solid and liquid bases *e.g.* Et_3_N (triethylamine), Na_2_CO_3_ (sodium carbonate), NaOEt (sodium ethoxide), and NaOH (sodium hydroxide), Na_2_CO_3_ as a suitable base provided the best conditions for the coupling of phenylboronic acid with C_6_H_5_–I in the presence of Pd-PO(BA)@SBA-15 catalyst at 80 °C.

**Table 2:** Optimization of different parameters for the synthesis of biphenyl using the Pd-PO(BA)@SBA-15 catalyst

Entry	Catalyst (mg)	Solvent	Base	Temperature (°C)	Time (min)	Yield (%)
1	10	PEG-400	Na_2_CO_3_	80	15	94
2	8	PEG-400	Na_2_CO_3_	80	30	89
3	12	PEG-400	Na_2_CO_3_	80	10	97
4	12	EtOH	Na_2_CO_3_	80	40	85
5	12	DMSO	Na_2_CO_3_	80	25	85
6	12	DMF	Na_2_CO_3_	80	70	80
7	12	H_2_O	Na_2_CO_3_	80	60	79
8	12	PEG-400	NaOH	80	40	84
9	12	PEG-400	NaOEt	80	55	80
10	12	PEG-400	Et_3_N	80	160	77
11	12	PEG-400	Na_2_CO_3_	60	45	80

To extend the scope of Pd-PO(BA)@SBA-15 performance, we studied a large number of aryl halides in the coupling reaction with phenylboronic acid (Table 3, entries 1–5). The obtained experimental results are outlined in Table 3, showing the high yield of the isolated biphenyl products and low reaction times in the coupling reaction of phenylboronic acid with aryl halides having electron-donating functional groups or electron-withdrawing functional groups. For example, Cl, OH, NO_2_, and Me functional groups on the aromatic ring of aryl halides were investigated.

**Table 3:** Synthesis of biphenyl derivatives in the presence Pd-PO(BA)@SBA-15

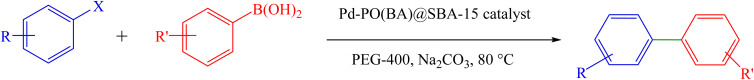								
Entry	R	R′	X	Product	Time (min)	Yield (%)	TOF (h^−1^)	Melting point (°C)
1	H	H	I	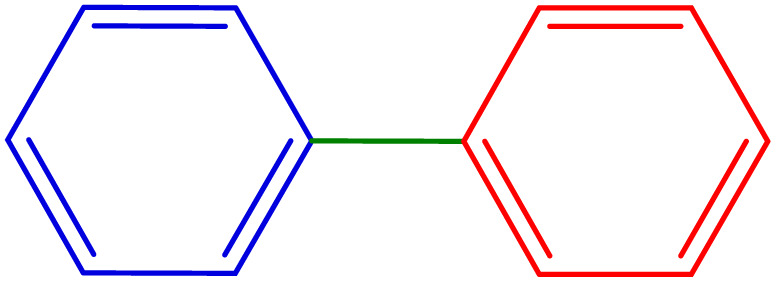	10	97	1353.5	64–67 (ref. 14)
2	4-NO_2_	H	Br	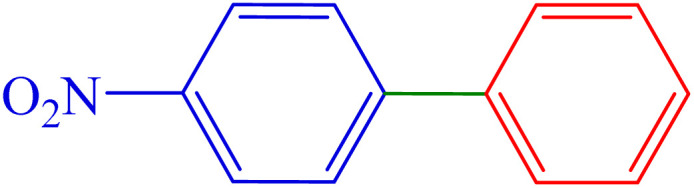	40	88	306.9	112–116 (ref. 14)
3	4-Cl	H	Br	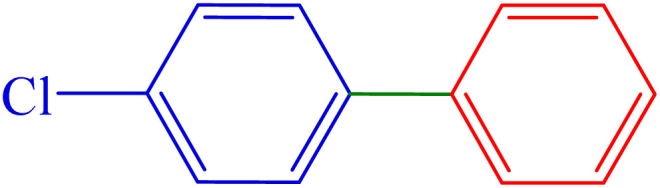	65	86	184.6	72–74 (ref. 14)
4	4-CN	H	Br	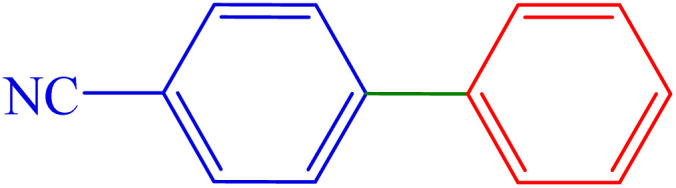	160	84	73.2	83–85 (ref. 14)
5	4-Me	H	I	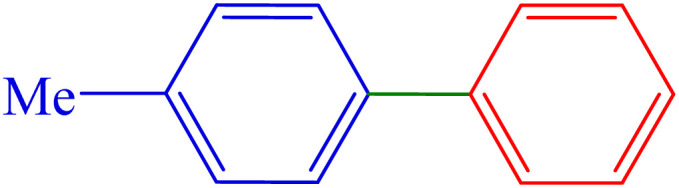	210	89	59.1	46–48 (ref. 14)
6	H	4-OMe	I	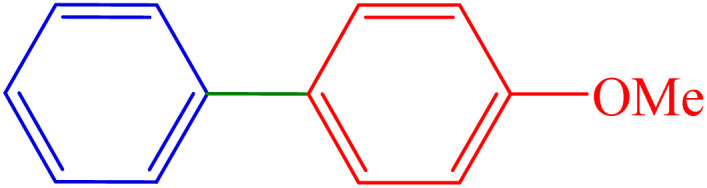	80	84	146.5	92–95 (ref. 15)
7	H	4-CHO	I	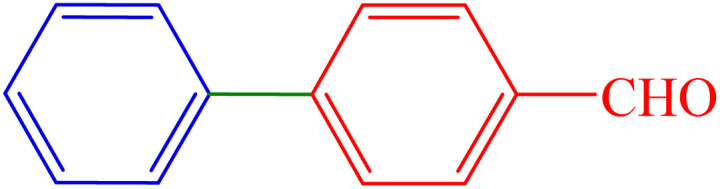	60	87	202.3	55–58 (ref. 14)
8	4-NO_2_	4-CHO	Br	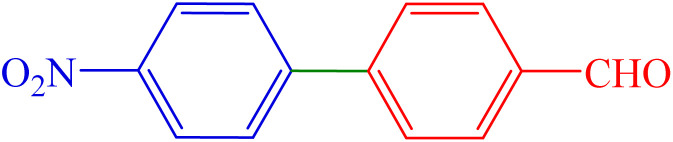	135	89	91.9	127–129 (ref. 10)
9	4-NO_2_	4-OMe	Br	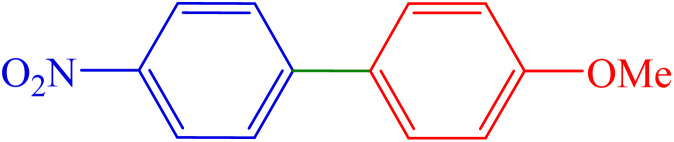	50	90	251.2	103–106 (ref. 10)

To extend the scope of Pd-PO(BA)@SBA-15 performance, except aryl halide derivatives, some derivatives of phenylboronic acid -having electron-donating functional groups or electron-withdrawing functional groups-were also investigated in the coupling reaction with aryl halides in the presence of this catalyst. For example, 4-methoxyphenylboronic acid was investigated in the Suzuki C–C coupling reaction with aryl iodides (Ar–I) and aryl bromides (Ar–Br) derivatives in the presence of Pd-PO(BA)@SBA-15 catalyst (Table 3, entries 6 and 9). Also, 4-formylphenylboronic acid was investigated in the Suzuki C–C coupling reaction with aryl iodides (Ar–I) and aryl bromides (Ar–Br) derivatives in the presence of Pd-PO(BA)@SBA-15 catalyst (Table 3, entries 7 and 8). All products were formed with high yield in the presence of Pd-PO(BA)@SBA-15 catalyst.

TOF (Turnover Frequency) and TON (Turnover Number) are important factors in determining the performance of catalysts.^12^ Therefore, TON is defined as moles of product/moles of catalyst, and TOF is defined as TON/reaction time (h).^13^ All biphenyl products were formed with high TOF and TON values in the presence of Pd-PO(BA)@SBA-15 catalyst, indicating the practicality of this catalyst.

**Scheme 5:**
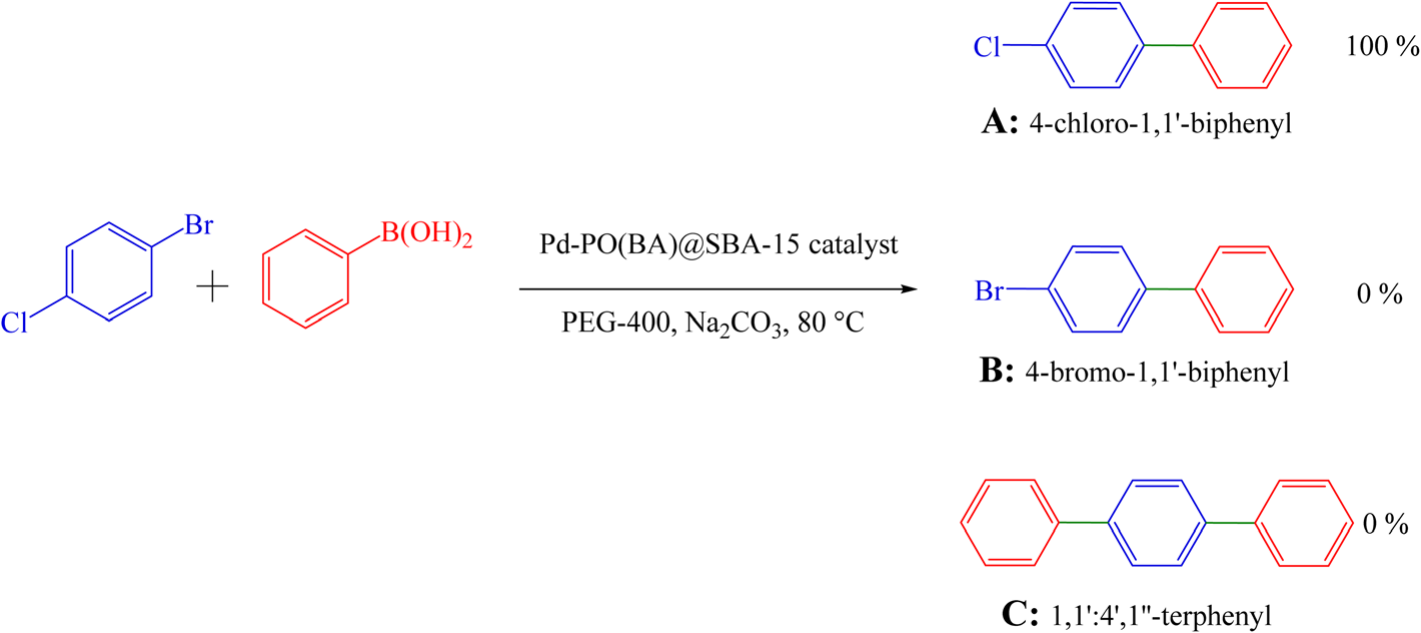
Selectivity of Pd-PO(BA)@SBA-15 catalyst in the Suzuki C–C coupling reaction.

Also, 1-chloro-4-bromobenzene was investigated for the formation of the biphenyl (Table 3, entry 3), indicating good selectivity of Pd-PO(BA)@SBA-15 catalyst in the Suzuki C–C coupling reaction (Scheme 5). The selectivity of Pd-PO(BA)@SBA-15 catalyst in the coupling reaction of phenylboronic acid with iodobenzene has been confirmed using the melting point. Melting points for possible products A (4-chloro-1,1′-biphenyl), B (4-bromo-1,1′-biphenyl) and C (1,1′ : 4′,1″-terphenyl) are reported as 71–73 °C, 89–92 °C and 212–213 °C, respectively. As shown in entry 3 (Table 3), the obtained melting point for the synthesized product in the presence of Pd-PO(BA)@SBA-15 catalyst is 72–74 °C, corresponding with product A.

The homoselectivity of Pd-PO(BA)@SBA-15 in the coupling reaction of phenylboronic acid with iodobenzene toward the synthesis of A, B, or C has been investigated using ^13^C NMR spectroscopy. In the ^13^C NMR spectrum of C, six peaks for carbons should be observed. But in the ^13^C NMR spectra of A and B, eight peaks for carbons should be observed. As can be seen in the ^13^C NMR spectrum of the final synthesized product, eight peaks have been observed, as follows: ^13^C NMR (100 MHz, CDCl_3_): 140.0, 139.7, 133.4, 128.9, 128.9, 128.4, 127.6, 127.0 ppm. So product C is not formed.

Therefore, based on the melting point and NMR data, only 4-chloro-1,1′-biphenyl is selectively formed in the presence of Pd-PO(BA)@SBA-15 catalyst.

**Scheme 6:**
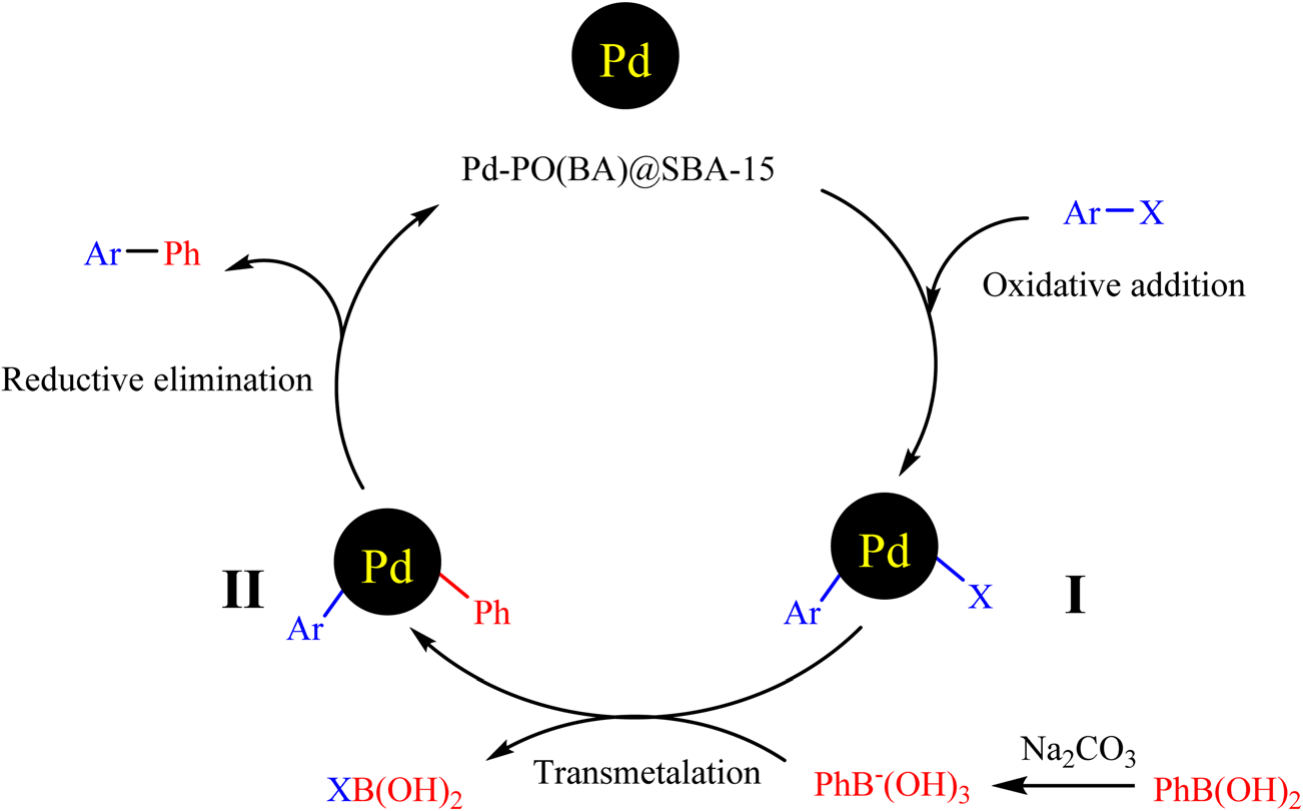
A catalytic cycle mechanism for the synthesizing of biphenyl using Suzuki C–C coupling reaction in the presence of Pd-PO(BA)@SBA-15 catalyst.

Scheme 6 investigates a mechanism for the C–C bond formation through the Suzuki coupling reaction in the presence of Pd-PO(BA)@SBA-15 catalyst.^1*b*^ First, the oxidative addition of aryl halide with Pd-complex on the surface of SBA-15 forms intermediate I. Then, an acid/base reaction occurs between aryl boronic acid and sodium carbonate. Then, intermediate II was formed by a transmetalation step. Finally, intermediate II with a reductive elimination is converted to the biphenyl product and regeneration of the catalyst, which returns in the catalytic cycle of the reaction.

#### Reusability of the Pd-PO(BA)@SBA-15 catalyst

**Fig. 6:**
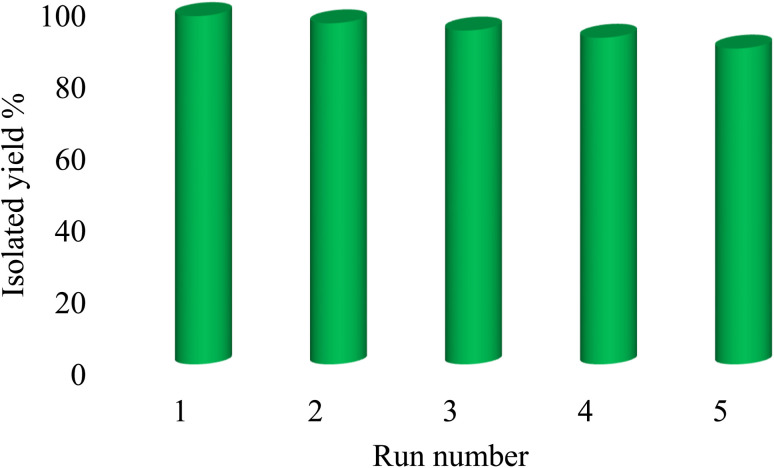
Pd-PO(BA)@SBA-15 catalyst recycling in the synthesizing of biphenyl.

In order to check the reusability of Pd-PO(BA)@SBA-15 catalyst, the coupling of phenylboronic acid with C_6_H_5_–I in the presence of Pd-PO(BA)@SBA-15 catalyst was repeated for the formation of the corresponding biphenyl under optimal reaction conditions. After the completion of the reaction, the Pd-PO(BA)@SBA-15 catalyst was isolated, and reused again in the next run. This experiment was repeated 5 times. As shown in Fig. 6, Pd-PO(BA)@SBA-15 catalyst was reused without a significant loss of its catalytic activity.

#### Comparison of the catalyst

Practicality of Pd-PO(BA)@SBA-15 catalyst for the synthesis of biphenyls in the C–C coupling reaction was compared with the other reported catalysts in Table 4. The coupling of phenylboronic acid with C_6_H_5_–I was selected for this comparison. Synthesis of biphenyl in the presence of other catalysts has been reported with low yield and time-consuming reactions. While the synthesis of biphenyl in the presence of Pd-PO(BA)@SBA-15 provides a 97% yield after 10 minutes, which is better than previous catalysts. In this comparison, the previous catalysts in entries 1–9 (Table 4) provided a longer reaction time or lower yield for the synthesis of biphenyl. In addition, the previous catalysts in entry 1 (Table 4) include a precious and rare metal as the main site of the catalyst. Synthesis of biphenyl in the presence of previous catalysts was reported in toxic organic solvents such as DMSO (Table 4, entry 4), DMF (Table 4, entries 2 and 6), THF (Table 4, entry 5) and 1,4-dioxane (Table 4, entry 3). However, the use of Pd-PO(BA)@SBA-15 catalyst does not have these disadvantages.

**Table 4:** Comparison of Pd-PO(BA)@SBA-15 for the synthesis of biphenyl with previously reported catalyst through coupling of phenylboronic acid with C_6_H_5_–I as a model reaction

Entry	Catalyst	Reaction conditions	Time (min)	Yield (%)	Ref.
1	Ru-dithizone@biochar-Ni MNPs	H_2_O, Na_2_CO_3_, 80 °C	90	96	16
2	Polymer anchored Pd(ii)	DMF/H_2_O, K_2_CO_3_, 80 °C	300	96	17
3	PANI-Pd	1,4-Dioxane : H_2_O (1 : 1), K_2_CO_3_, 95 °C	240	91	18
4	ZnFe_2_O_4_@SiO_2_@CPTMS@PYA-Pd	DMSO, K_2_CO_3_, 95 °C	100	96	4
5	NHC–Pd(ii) complex	THF, CsCO_3_, 80 °C	12 h	88	19
6	Pd(ii)–NHC complex	DMF, CsCO_3_, 100 °C	24 h	99	20
7	Cu–ninhydrin@GO–Ni MNPs	H_2_O, Na_2_CO_3_, 80 °C	50	97	21
8	CA/Pd(0)	H_2_O, K_2_CO_3_, 100 °C	120	94	22
9	Pd NP	H_2_O, K_2_CO_3_, 100 °C	12h	95	23
10	Pd-PO(BA)@SBA-15	PEG-400, Na_2_CO_3_, 80 °C	10	97	This work

## Conclusion

In summary, we synthesized the SBA-15 as a support, then its surface was modified using the IPTES ligand. Then the modified SBA-15 was functionalized by 2,2′-(propane-1,3-diylbis(oxy))dibenzenaminium chloride ligand (PO(BA)) for immobilization of palladium as an efficient nanocatalyst for organic reactions such as the Suzuki C–C coupling reaction. The synthesized Pd-PO(BA)@SBA-15 catalyst was characterized by SEM, ICP, TGA/DSC EDS, BET/BJH, and WDX techniques. TGA analysis showed thermal stability of this catalyst up to 230 °C. BET/BJH methods showed a high surface area for this catalyst, indicating suitable application as a practical catalyst. Pd-PO(BA)@SBA-15 was investigated as a friendly environmental catalyst in the synthesis of biphenyl derivatives. All products were successfully formed with high yields and high TOF values in very fast reaction rates in PEG-400 as a green and available solvent. Pd-PO(BA)@SBA-15 exhibited a good selectivity in the synthesis of biphenyl derivatives. In this work, various aryl halide (Ar–X) derivatives (having electron-donating functional or electron-withdrawing functional groups) were successfully coupled with various aryl boronic acid derivatives (such as phenylboronic acid, 4-methoxyphenylboronic acid and 4-formylphenylboronic acid). Also, Pd-PO(BA)@SBA-15 was reused 5 times without reducing its activity.

## Conflicts of interest

There are no conflict of declare.

## Supplementary Material

RA-016-D5RA08145A-s001

## Data Availability

All data generated or analyzed during this study are included in this published article and supplementary information (SI). Supplementary information: NMR data. See DOI: https://doi.org/10.1039/d5ra08145a.
