# Supplementary material for: A palladium complex of 2,2′-(propane-1,3-diylbis(oxy))dibenzenaminium chloride on SBA-15 as a returnable, environmental and selective nanostructured-catalyst in the Suzuki C–C coupling reaction
Source: RSC Adv. 2026 Apr 27;16(24):21896–907. doi: 10.1039/d5ra08145a (PMC13118169; doi:10.1039/d5ra08145a)

Supplementary Information (SI) for RSC Advances.
This journal is © The Royal Society of Chemistry 2026

Supplementary Data

A palladium complex of 2,2'-(propane-1,3-diylbis(oxy))dibenzenaminium chloride on SBA-15 as a returnable, environmental and selective nanostructured-catalyst in the Suzuki C-C coupling reaction

Shirin Mohammadi, Mohsen Nikoorazm*, Bahman Tahmasbi*, Yunes Abbasi Tyula

Department of Chemistry, Faculty of Science, Ilam University, P. O. Box 69315516, Ilam, Iran.

E-mail addresses: m.nikorazm@ilam.ac.ir (M. Nikoorazm), b.tahmasbi@ilam.ac.ir

ABSTRACT

In this work, mesoporous SBA-15 was synthesized by a simple procedure using P123 and TEOS in HCl acidic conditions, followed by calcination at 550 ºC. 2,2'-(propane-1,3-diylbis(oxy))dibenzenaminium chloride ligand (PO(BA)) as ligand was synthesized from 2-nitrophenol and 3,1-dibromopropane, following reduction of nitro groups. The step-by-step synthesis of the PO(BA) ligand was confirmed by ^{1}H NMR and ^{13}C NMR. 3-Iodopropyltriethoxysilane (IPTES) was synthesized from 3-chloropropyltriethoxysilane (CPTES) in dry acetone. Then, the mesoporous SBA-15 surface was modified by IPTES and next it was functionalized by PO(BA). Finally, immobilized PO(BA) on SBA-15 became complex with palladium acetate (Pd-PO(BA)@SBA-15). The prepared Pd-PO(BA)@SBA-15 was characterized by SEM, ICP, TGA/DSC EDS, BET/BJH, and WDX techniques. TGA and BET/BJH methods showed high thermal stability of this catalyst up to 230 ºC and a high surface area for this catalyst. Then, the catalytic usage of Pd-PO(BA)@SBA-15 was investigated in the selective carbon-carbon bond formation. Various aryl halides (including aryl iodides (Ar-I) and aryl bromides (Ar-Br), having electron-donating or electron-withdrawing functional groups) and some derivatives of phenylboronic acid (such as phenylboronic acid ($C_6H_5(OH)_2$), 4-methoxyphenylboronic acid and 4-formylphenylboronic acid) were investigated and all biphenyl products were obtained with high yields and TOF values. NMR spectroscopy was used to determine the synthesized biphenyl products. Pd-PO(BA)@SBA-15 catalyst was shown to be reusable without significant loss in its performance in the Suzuki-Miyaura cross-coupling reaction.

Keywords: Mesoporous SBA-15, selective nanocatalyst, C-C coupling reaction, palladium complex, reusable catalyst

NMR spectral data

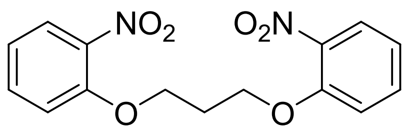

1,3-bis(2-nitrophenoxy)propane

^{1}H NMR (250 MHz, δ ppm DMSO-d_6): 7.84 (d, *J*= 7.5 Hz, 2H), 7.62 (t, *J*= 7.5 Hz, 2H), 7.34 (d, *J*= 7.5 Hz, 2H), 7.08 (t, *J*= 7.5 Hz, 2H), 4.31 (t, *J*= 7.5 Hz, 4H), 2.20 (quin, *J*= 7.5 Hz, 2H) ppm.

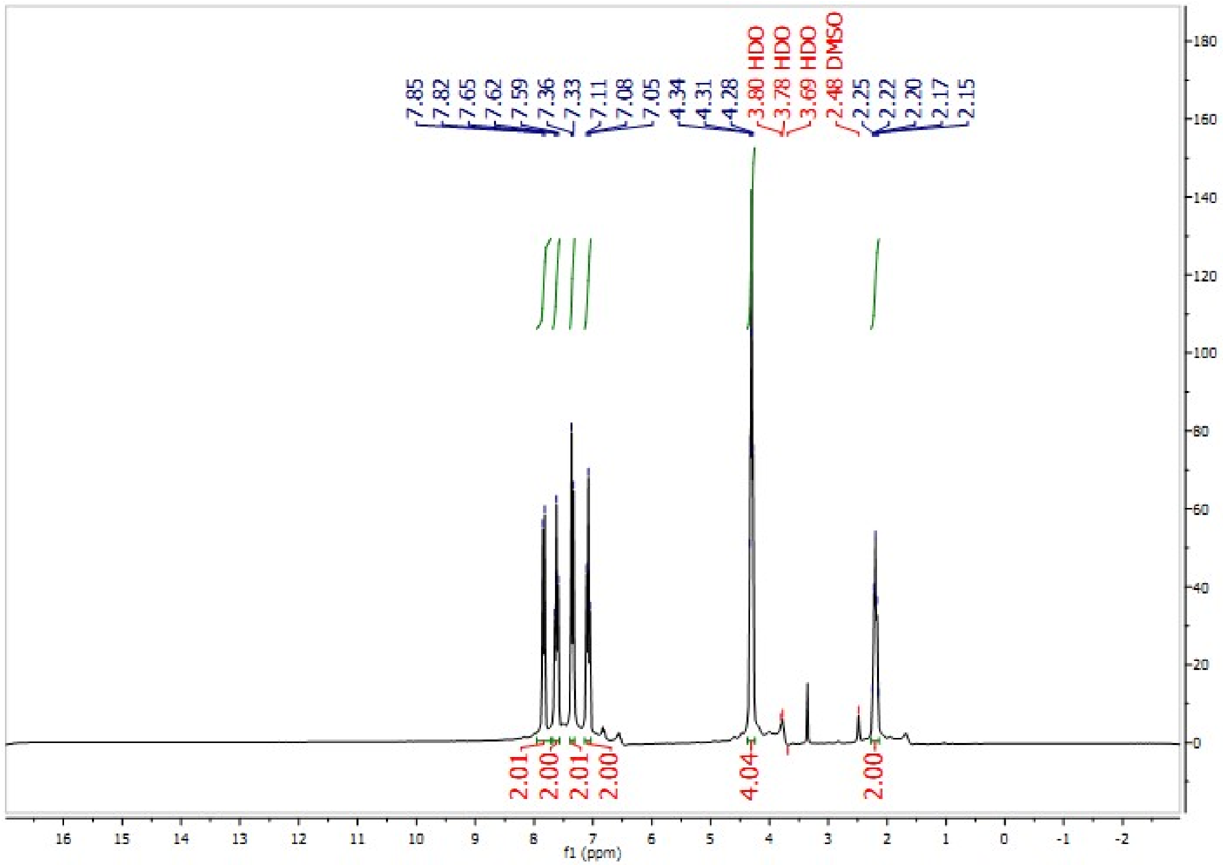

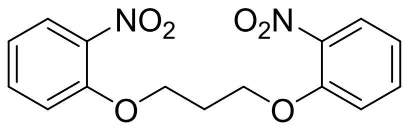

1,3-bis(2-nitrophenoxy)propane

^{13}C NMR (100 MHz, DMSO-d_6): 151.6, 139.9, 134.9, 125.4, 121.0, 115.4, 65.9, 28.5 ppm.

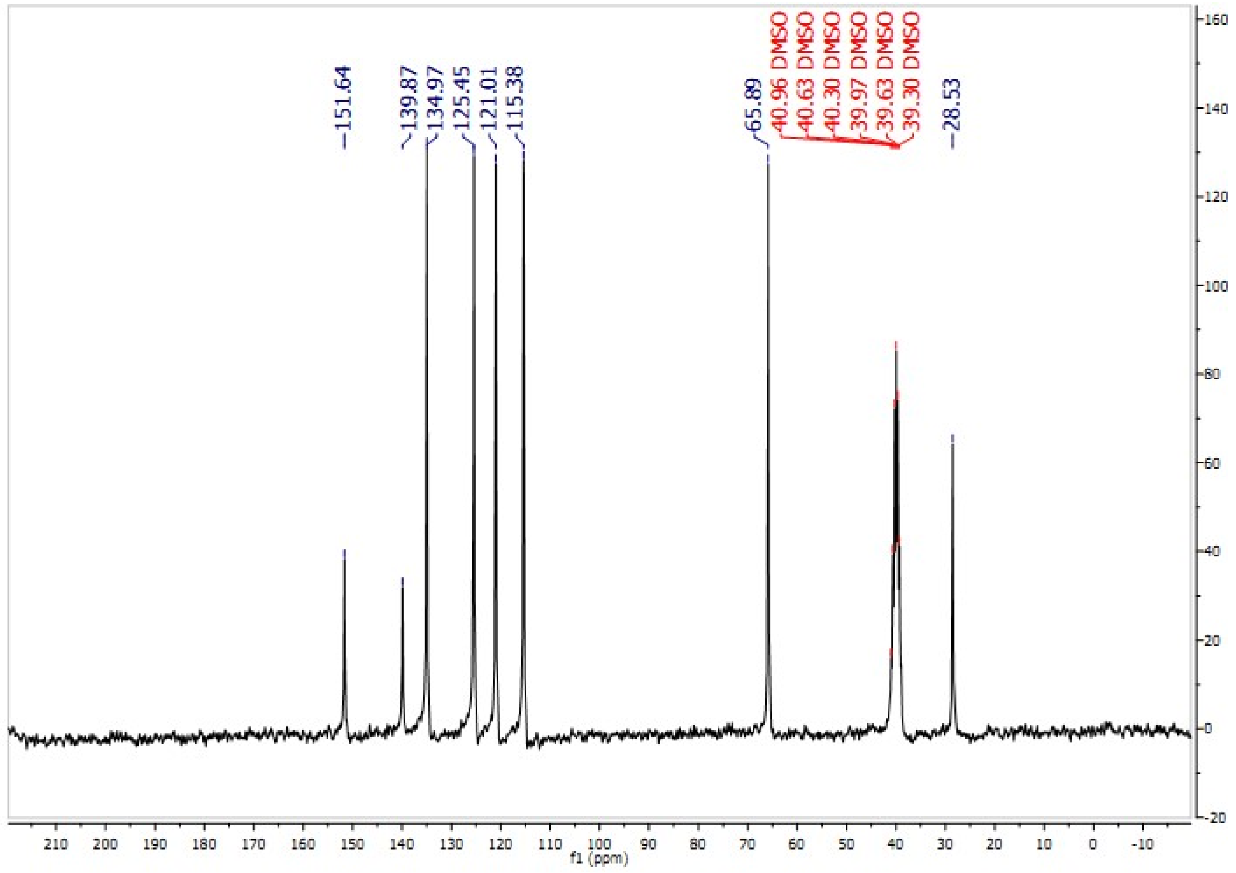

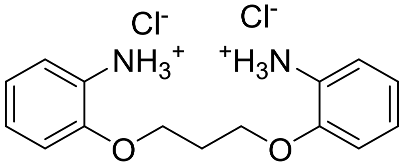

2,2'-(propane-1,3-diylbis(oxy))dibenzenaminium chloride

^{1}H NMR (250 MHz, δ ppm DMSO-d_6): 7.42-7.38 (m, 2H), 7.36-7.32 (m, 2H), 7.22 (d, *J*= 7.5 Hz, 2H), 7.00 (t, *J*= 7.5 Hz, 2H), 4.33 (t, *J*= 5 Hz, 4H), 3.80 (br, 6H), 2.23 (s, 2H) ppm.

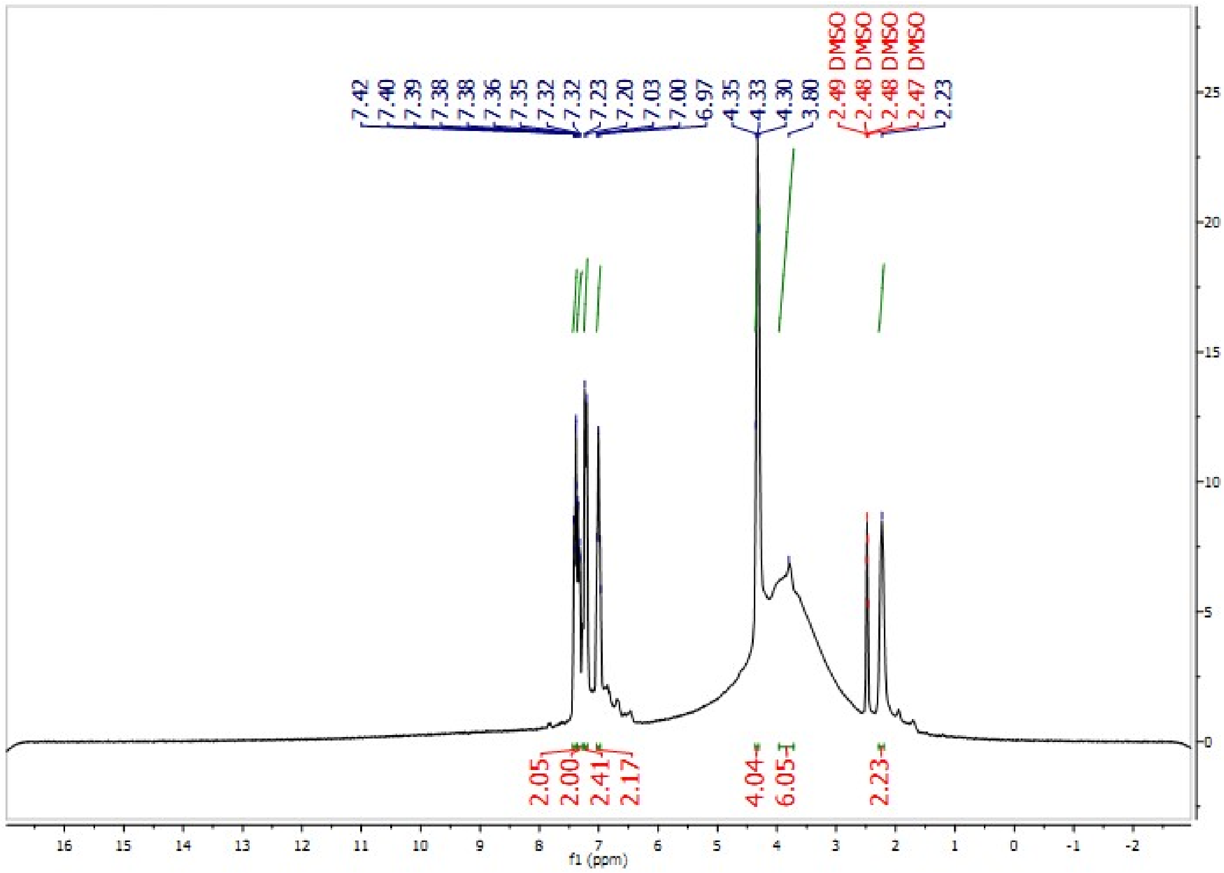

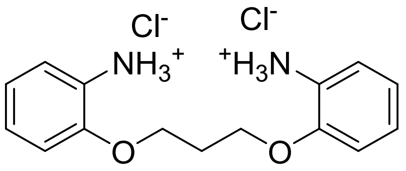

2,2'-(propane-1,3-diylbis(oxy))dibenzenaminium chloride

^{13}C NMR (100 MHz, DMSO-d$_6$): 151.8, 129.7, 124.2, 123.4, 121.0, 113.6, 65.5, 28.7 ppm.

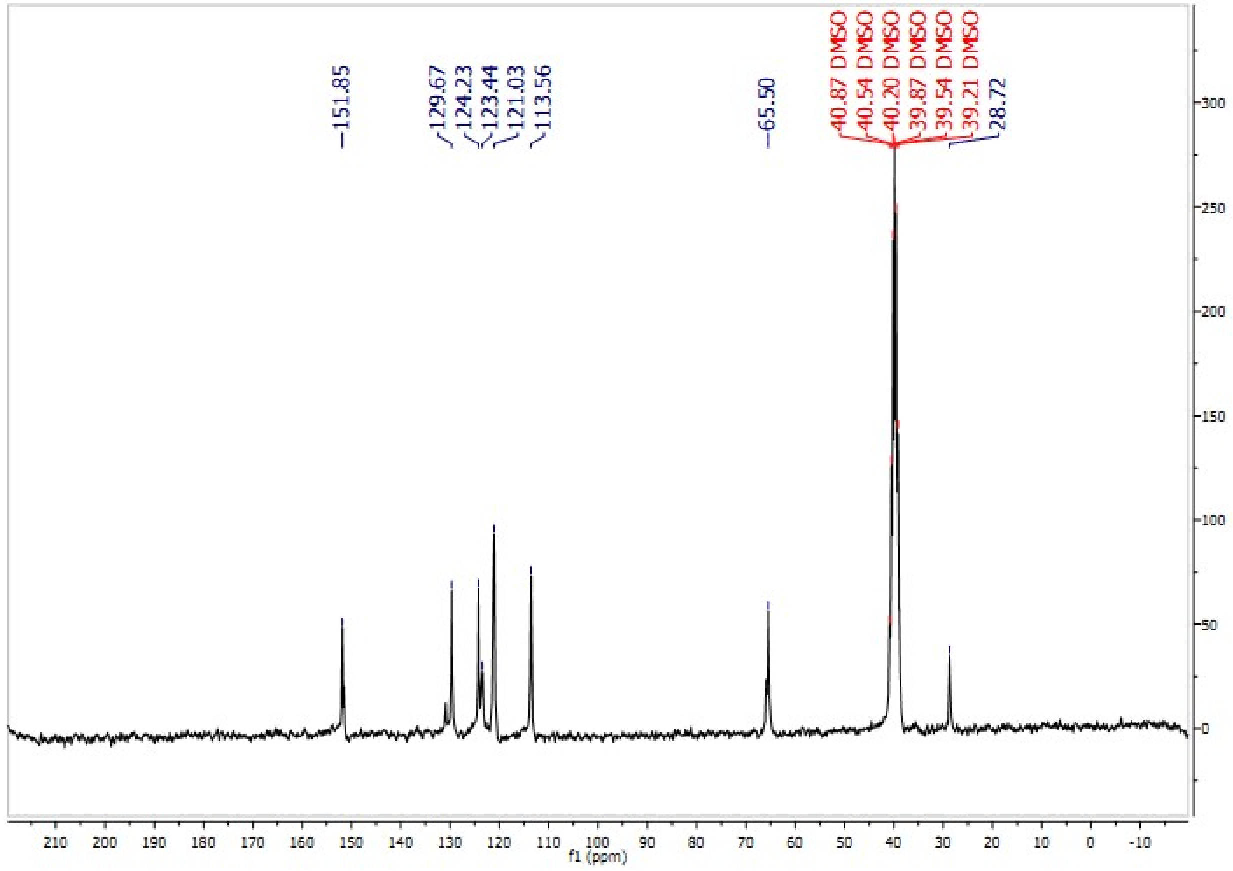

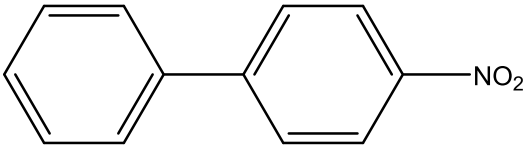

4-nitro-1,1'-biphenyl

^{1}H NMR (300 MHz, δ ppm DMSO-d_6): 8.29 (d, *J*= 9 Hz, 2H), 7.95 (d, *J*= 9 Hz, 2H), 7.77 (d, *J*= 6 Hz, 2H), 7.56-7.46 (m, 3H) ppm.

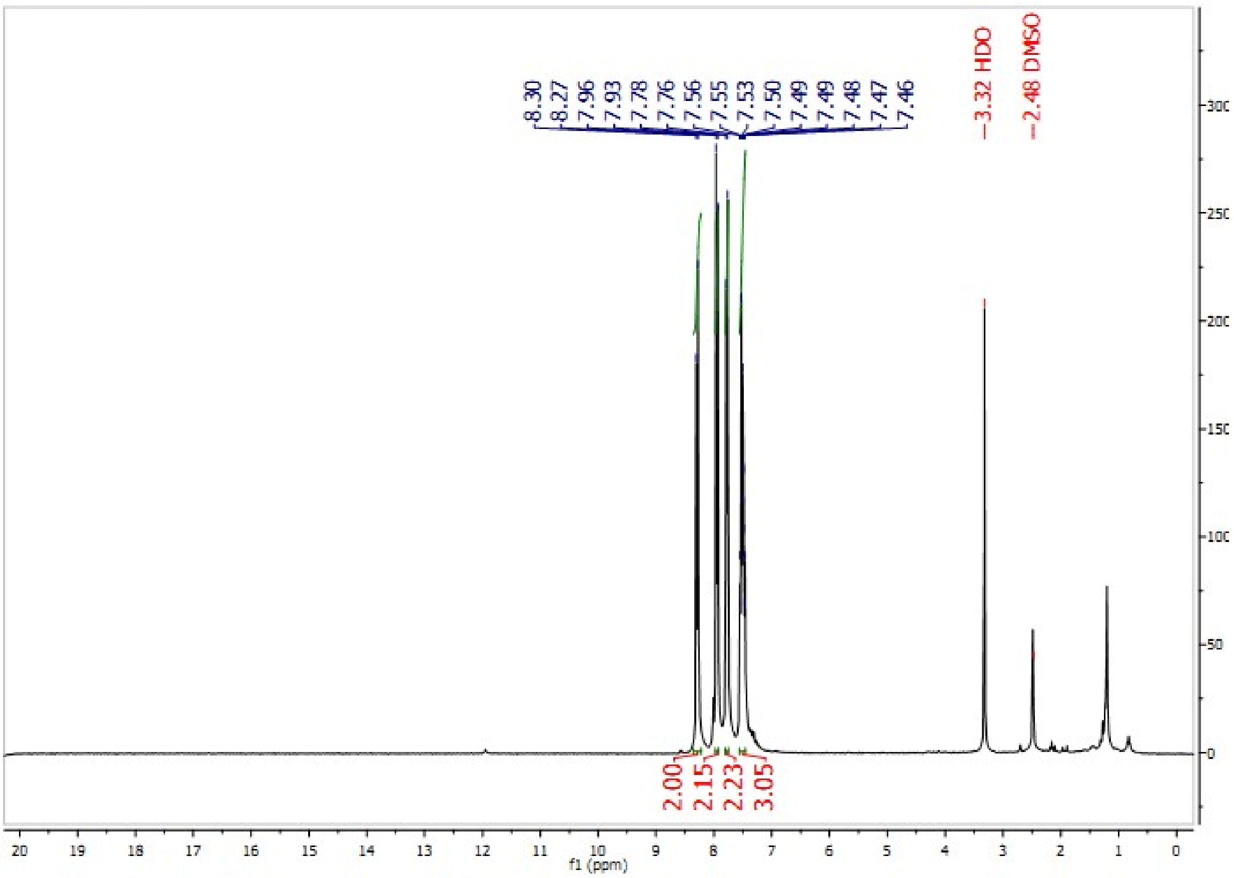

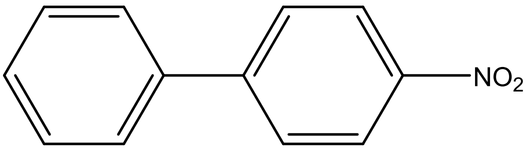

4-nitro-1,1'-biphenyl

^{13}C NMR (100 MHz, $CDCl_3$): 147.6, 147.1, 138.8, 129.2, 128.9, 127.8, 127.4, 124.1 ppm.

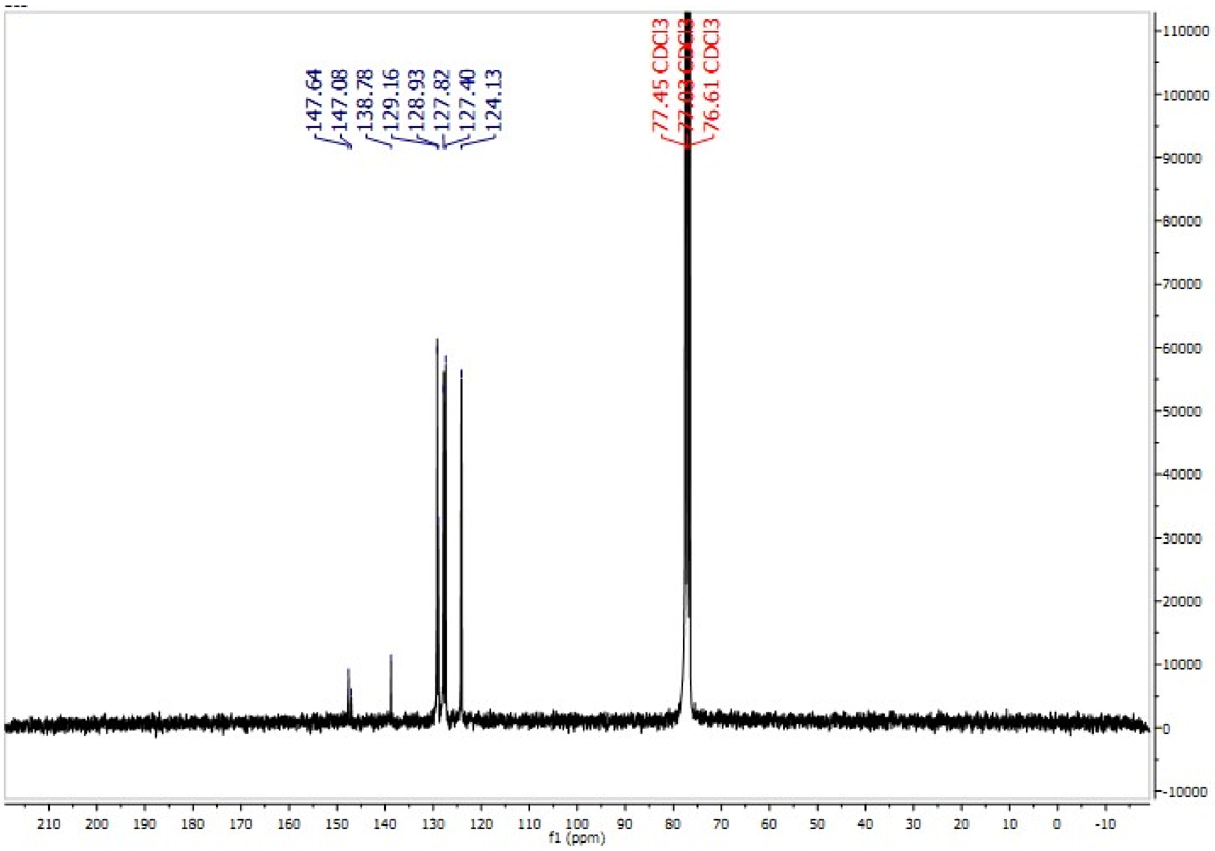

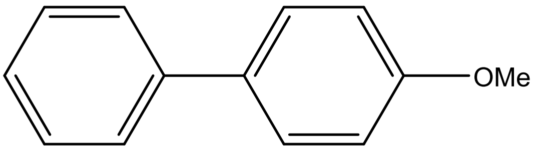

4-methoxy-1,1'-biphenyl

^{1}H NMR (300 MHz, δ ppm DMSO-d_6): 7.59 (d, *J*= 6 Hz, 4H), 7.41 (t, *J*= 7.5 Hz, 2H), 7.29 (t, *J*= 7.5 Hz, 1H), 7.01 (d, *J*= 9 Hz, 2H), 3.78 (s, 3H) ppm.

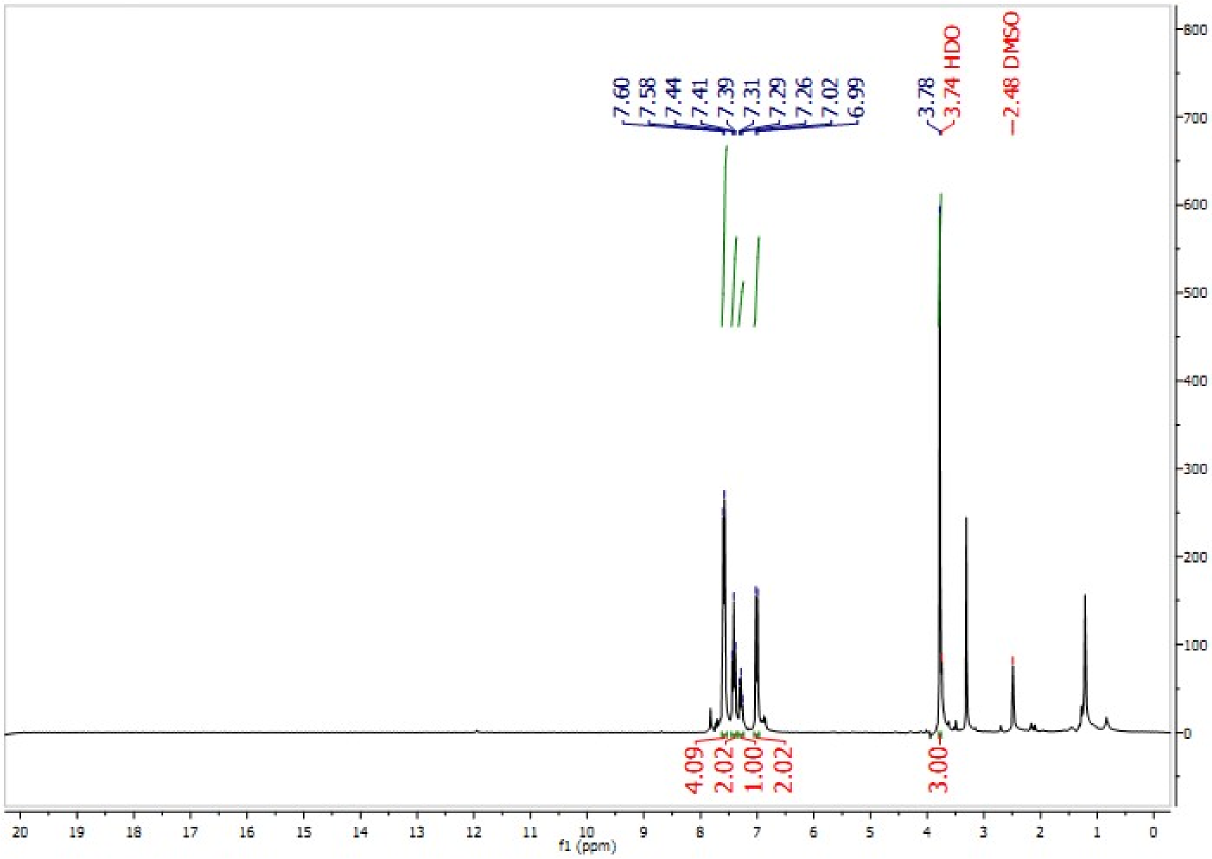

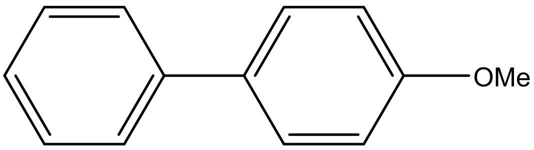

4-methoxy-1,1'-biphenyl

^{13}C NMR (100 MHz, $CDCl_3$): 159.1, 140.8, 133.8, 128.7, 128.2, 126.8, 126.7, 114.2, 55.4 ppm.

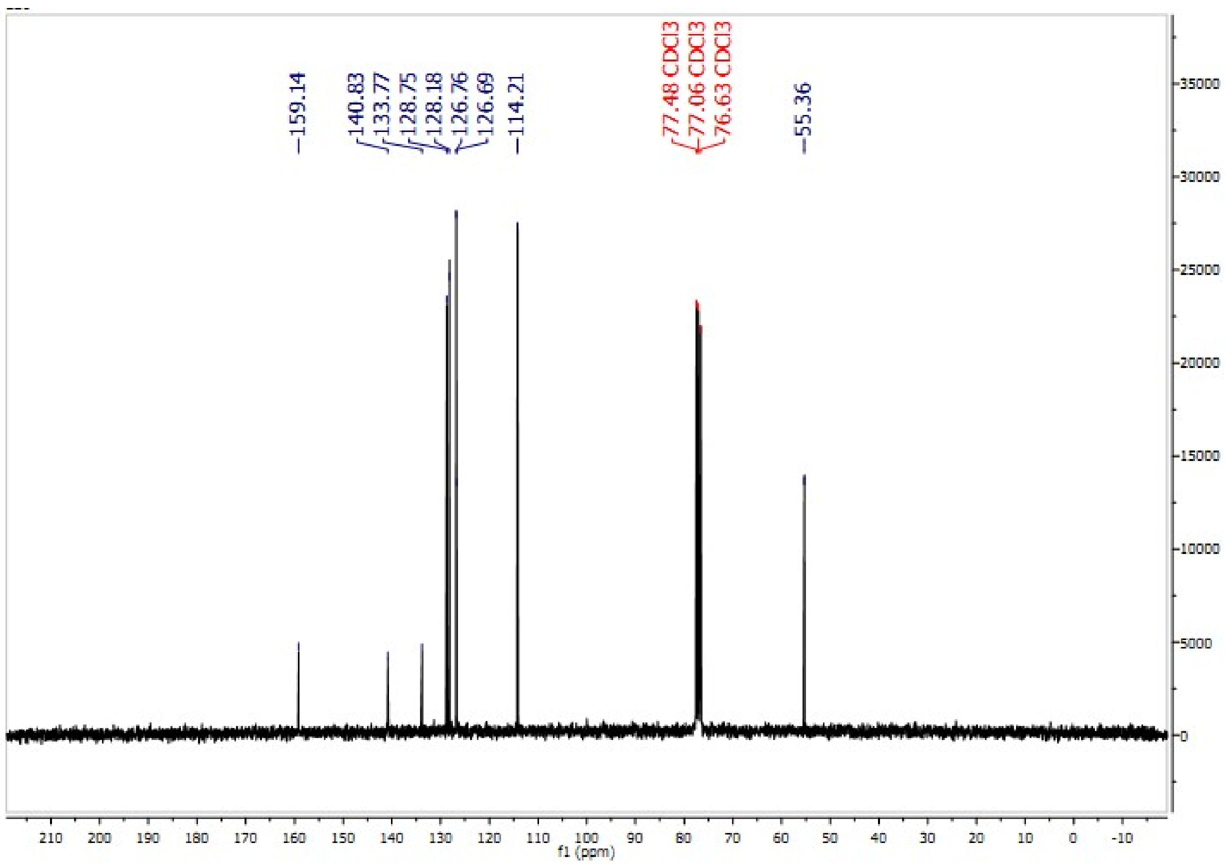

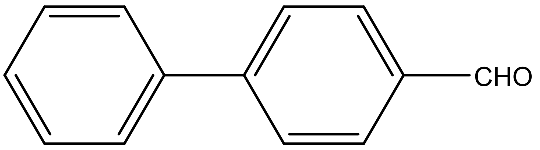

[1,1'-biphenyl]-4-carbaldehyde

^{1}H NMR (300 MHz, δ ppm DMSO-d_6): 10.04 (s, 1H), 7.98 (d, *J*= 9 Hz, 2H), 7.88 (t, *J*= 7.5 Hz, 2H), 7.75 (d, *J*= 6 Hz, 2H), 7.53-7.40 (m, 3H) ppm.

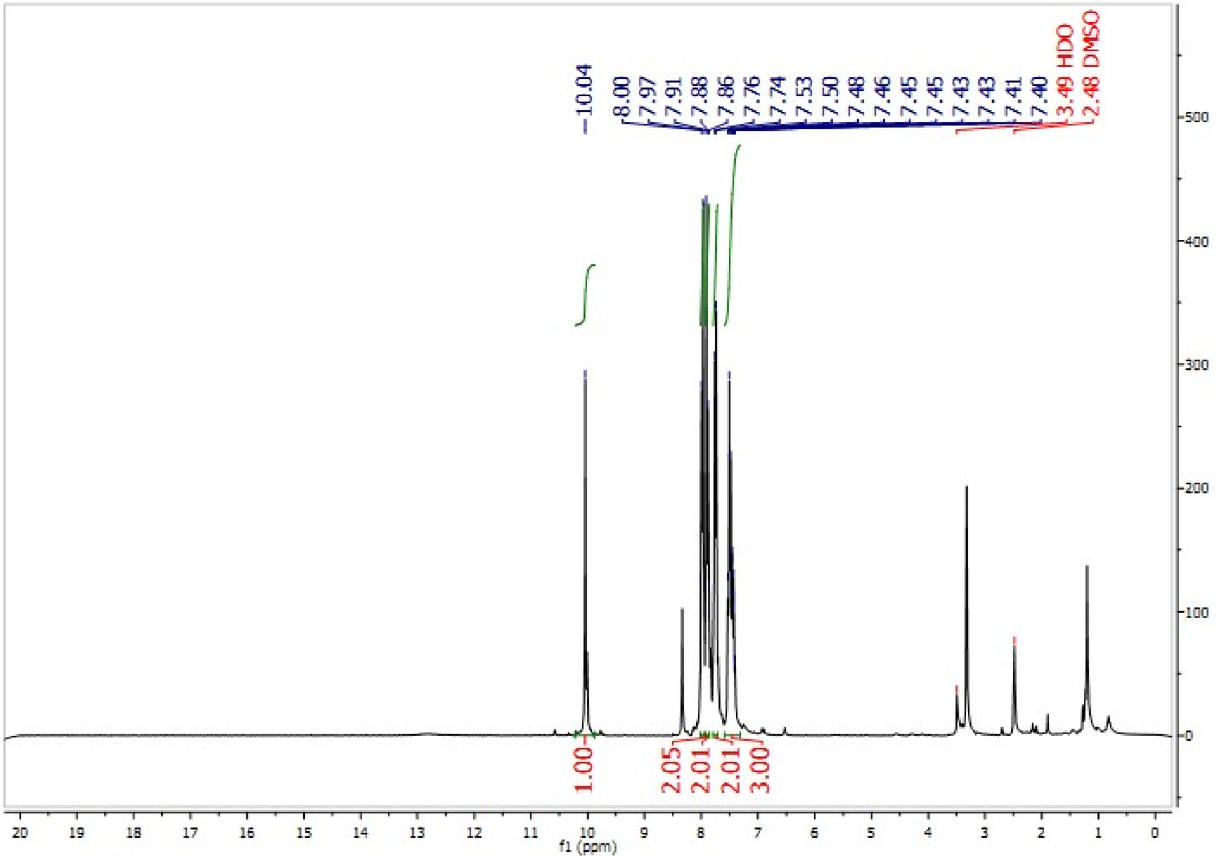

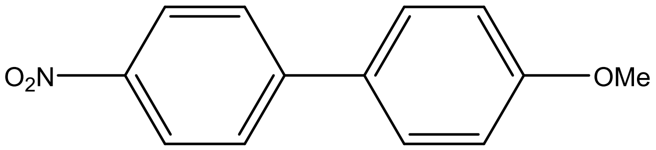

4-methoxy-4'-nitro-1,1'-biphenyl

^{1}H NMR (300 MHz, δ ppm DMSO-d_6): 8.25 (d, *J*= 9 Hz, 2H), 7.90 (d, *J*= 9 Hz, 2H), 7.75 (d, *J*= 9 Hz, 2H), 7.07 (d, *J*= 9 Hz, 2H), 3.81 (s, 3H) ppm.

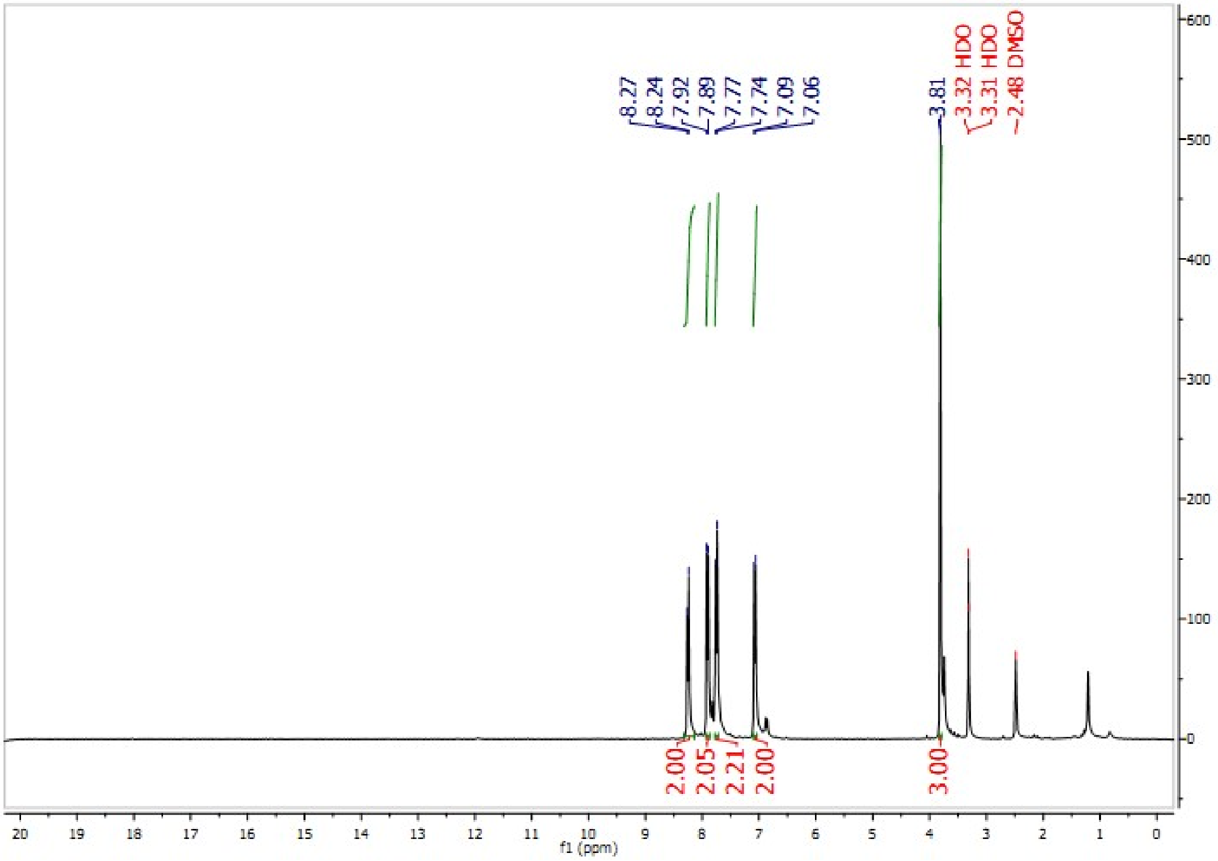

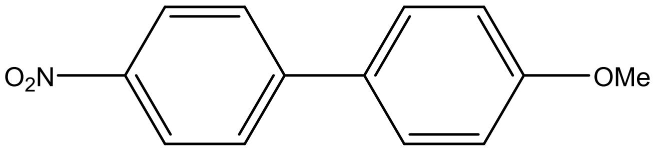

4-methoxy-4'-nitro-1,1'-biphenyl

^{13}C NMR (100 MHz, $CDCl_3$): 160.4, 147.2, 146.5, 131.0, 128.6, 127.1, 124.1, 114.6, 55.4 ppm.

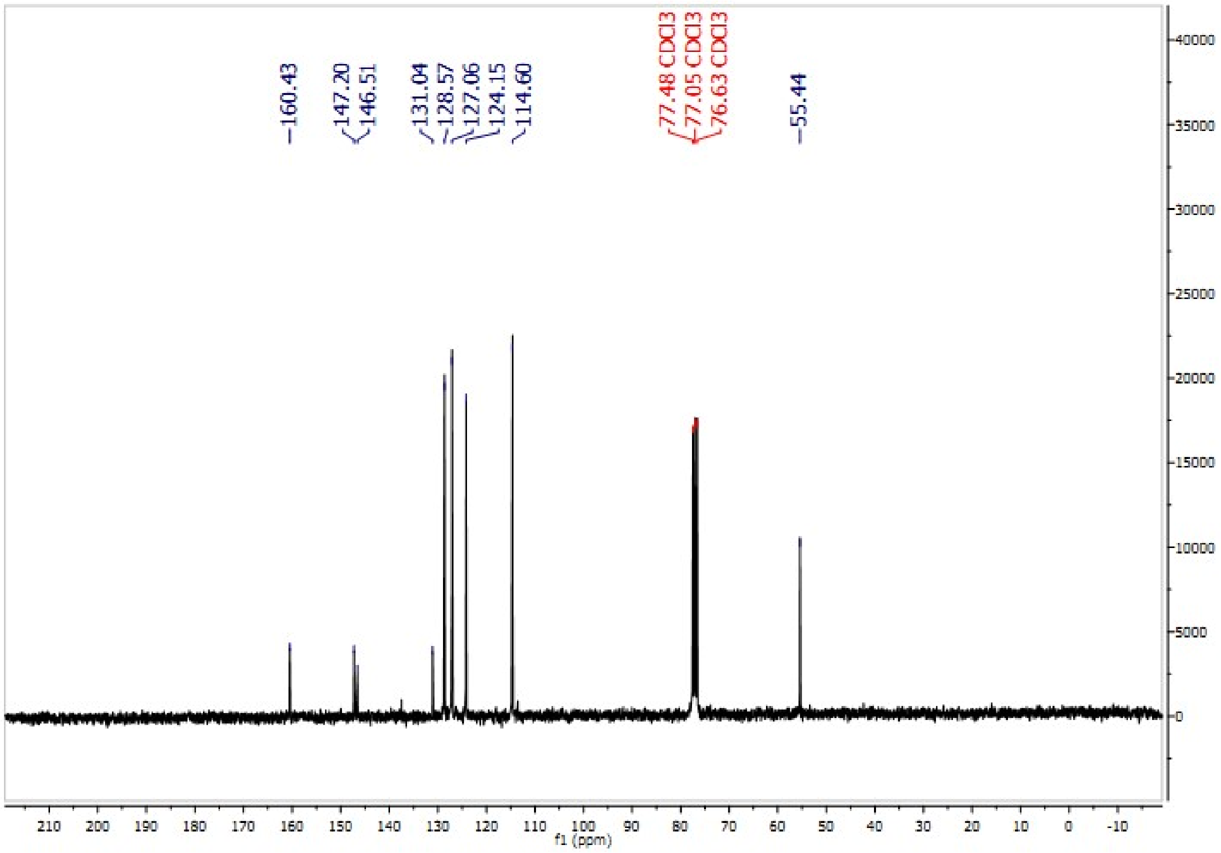

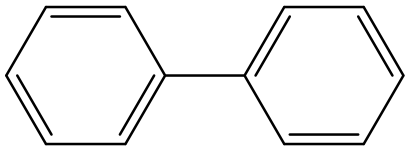

1,1'-biphenyl

^{1}H NMR (300 MHz, δ ppm $CDCl_3$): 7.63 (d, *J*= 9 Hz, 4H), 7.48 (t, *J*= 6 Hz, 4H), 7.39 (d, *J*= 9 Hz, 2H) ppm.

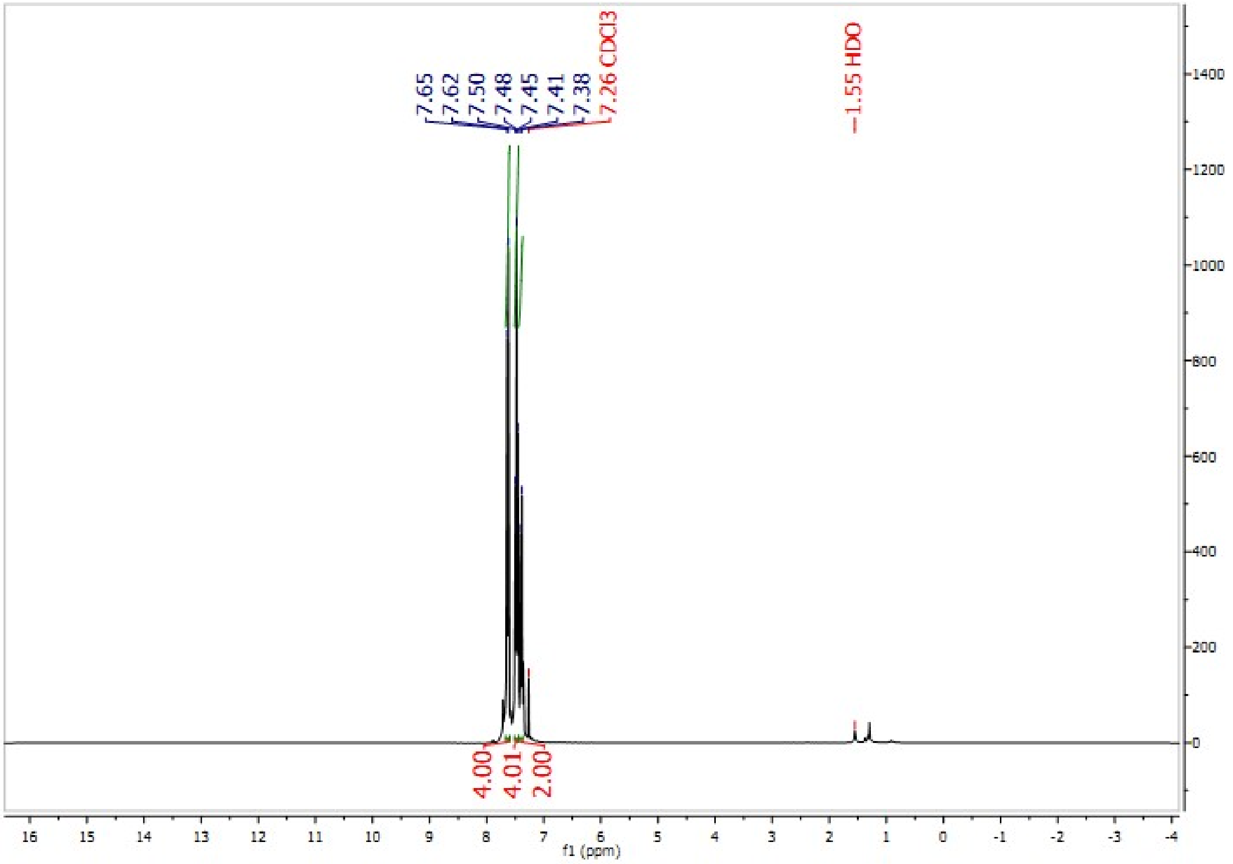

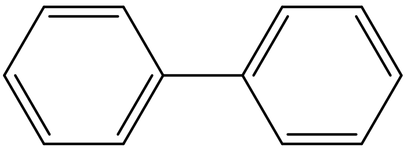

1,1'-biphenyl

^{13}C NMR (100 MHz, $CDCl_3$): 141.3, 128.8, 127.3, 127.2 ppm.

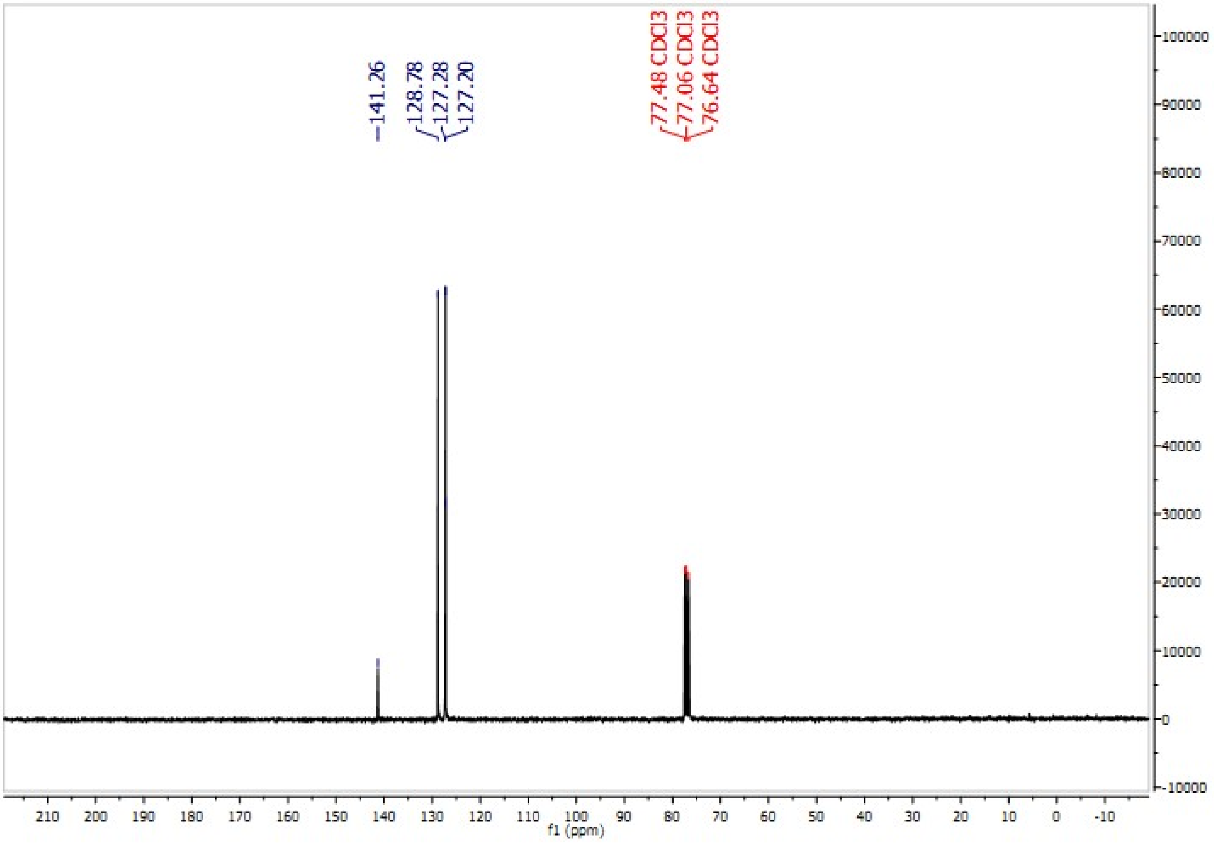

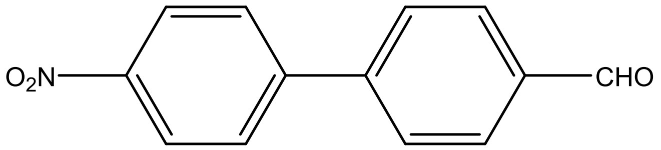

4'-nitro-[1,1'-biphenyl]-4-carbaldehyde

^{1}H NMR (300 MHz, δ ppm $CDCl_3$): 10.09 (s, 1H), 8.34 (d, *J*= 9 Hz, 2H), 8.02 (d, *J*= 9 Hz, 2H), 7.80 (d, *J*= 9 Hz, 4H) ppm.

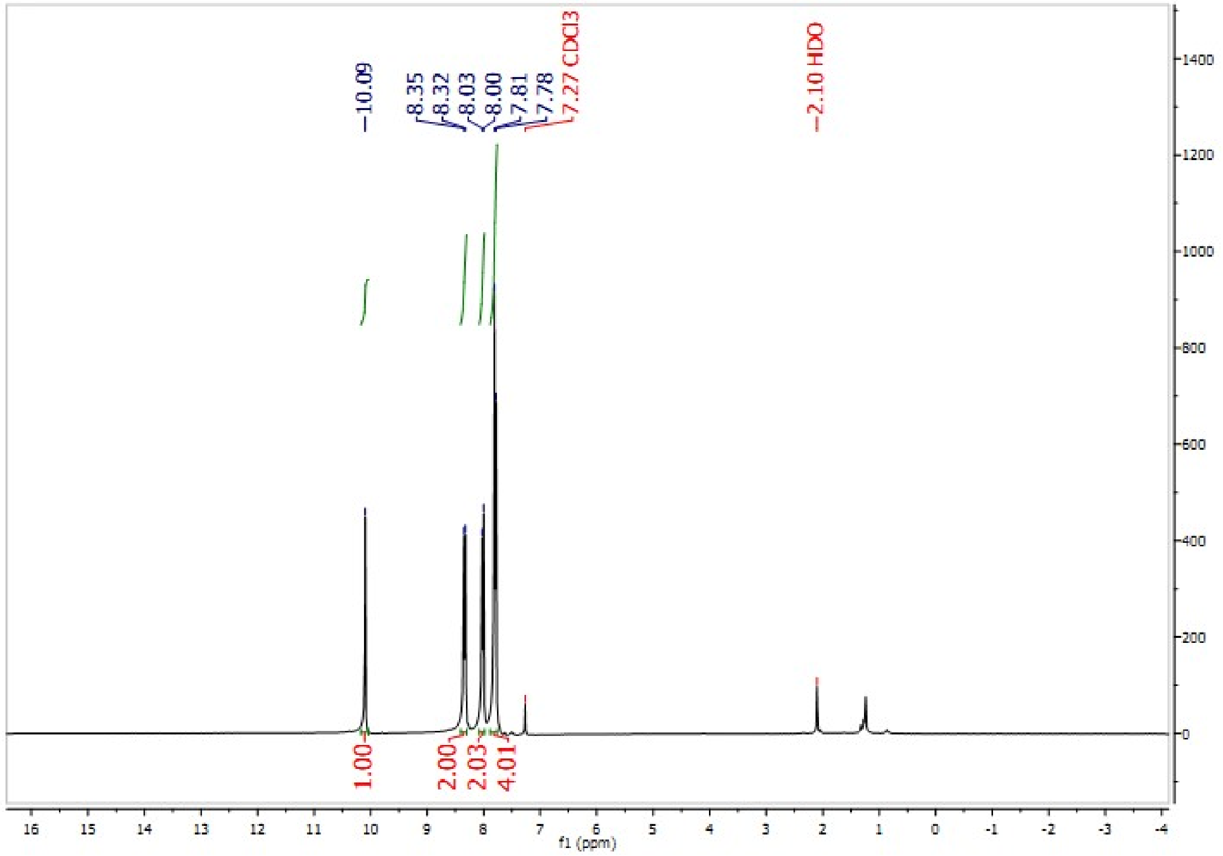

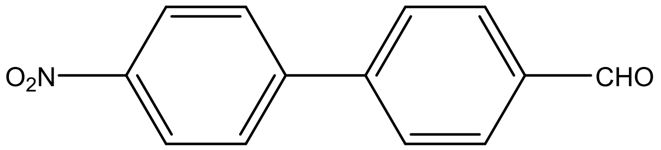

4'-nitro-[1,1'-biphenyl]-4-carbaldehyde

^{13}C NMR (100 MHz, $CDCl_3$): 191.7, 147.7, 146.0, 144.5, 136.2, 130.5, 128.2, 128.1, 124.3 ppm.

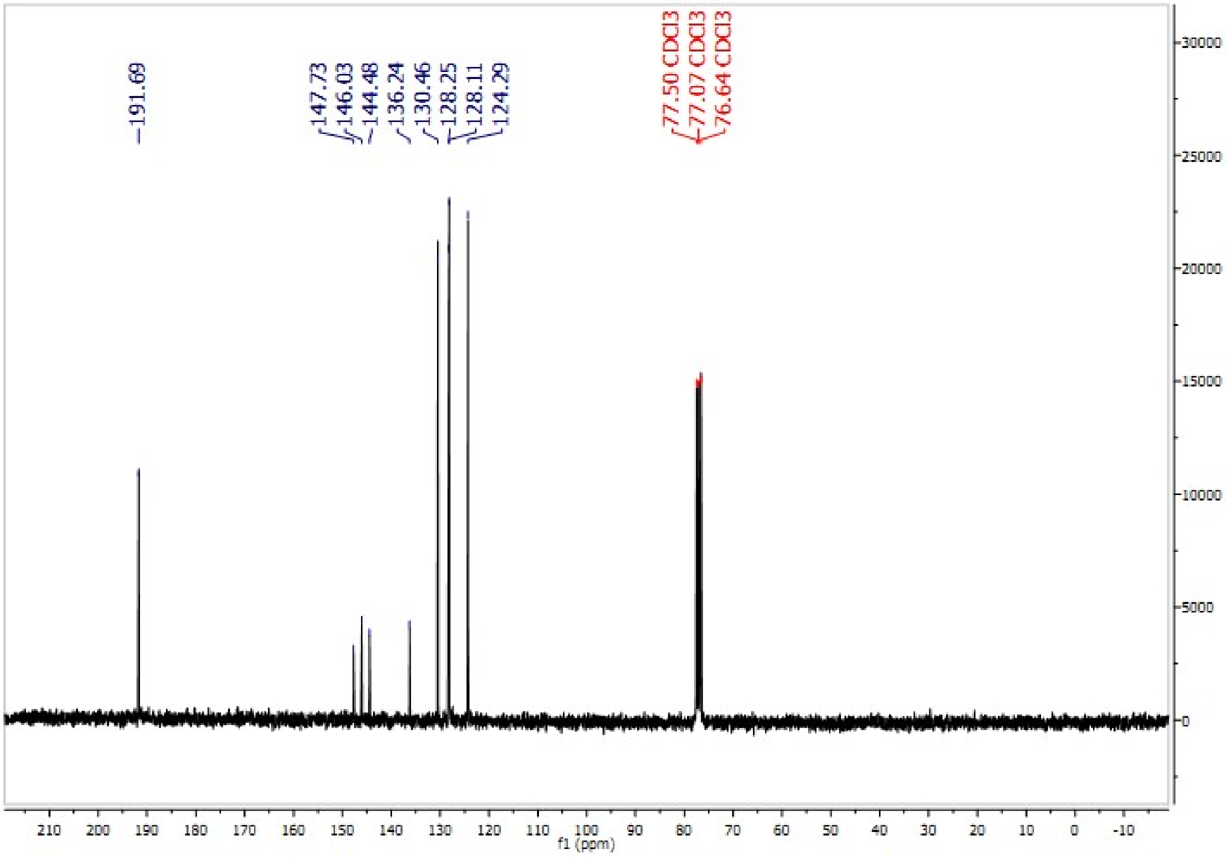

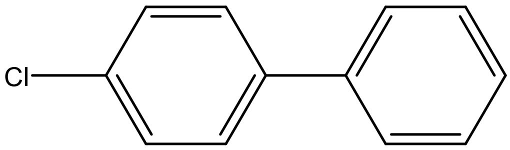

4-chloro-1,1'-biphenyl

^{1}H NMR (300 MHz, δ ppm $CDCl_3$): 7.58-7.52 (m, 4H), 7.46 (t, *J*= 9 Hz, 4H), 7.40 (t, *J*= 3 Hz, 1H) ppm.

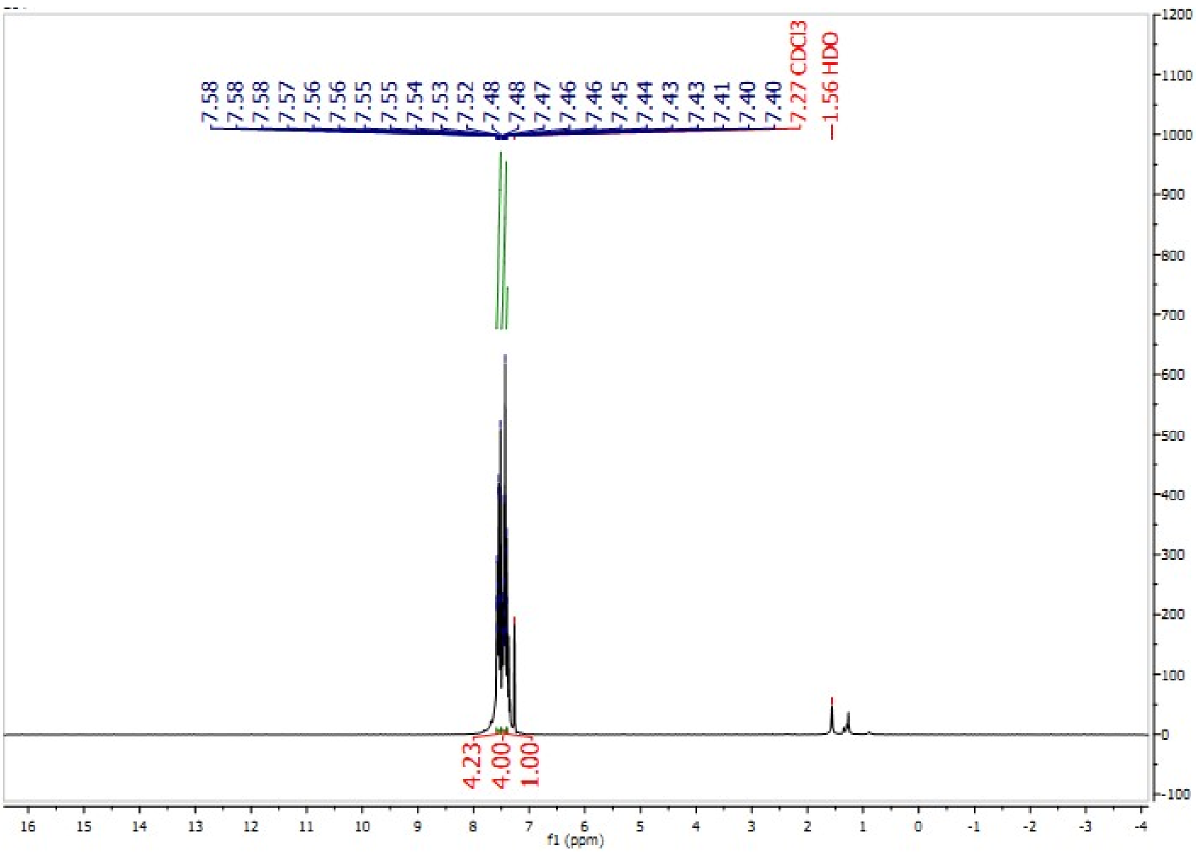

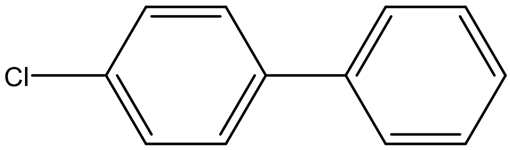

4-chloro-1,1'-biphenyl

^{13}C NMR (100 MHz, $CDCl_3$): 140.0, 139.7, 133.4, 128.9, 128.9, 128.4, 127.6, 127.0 ppm.

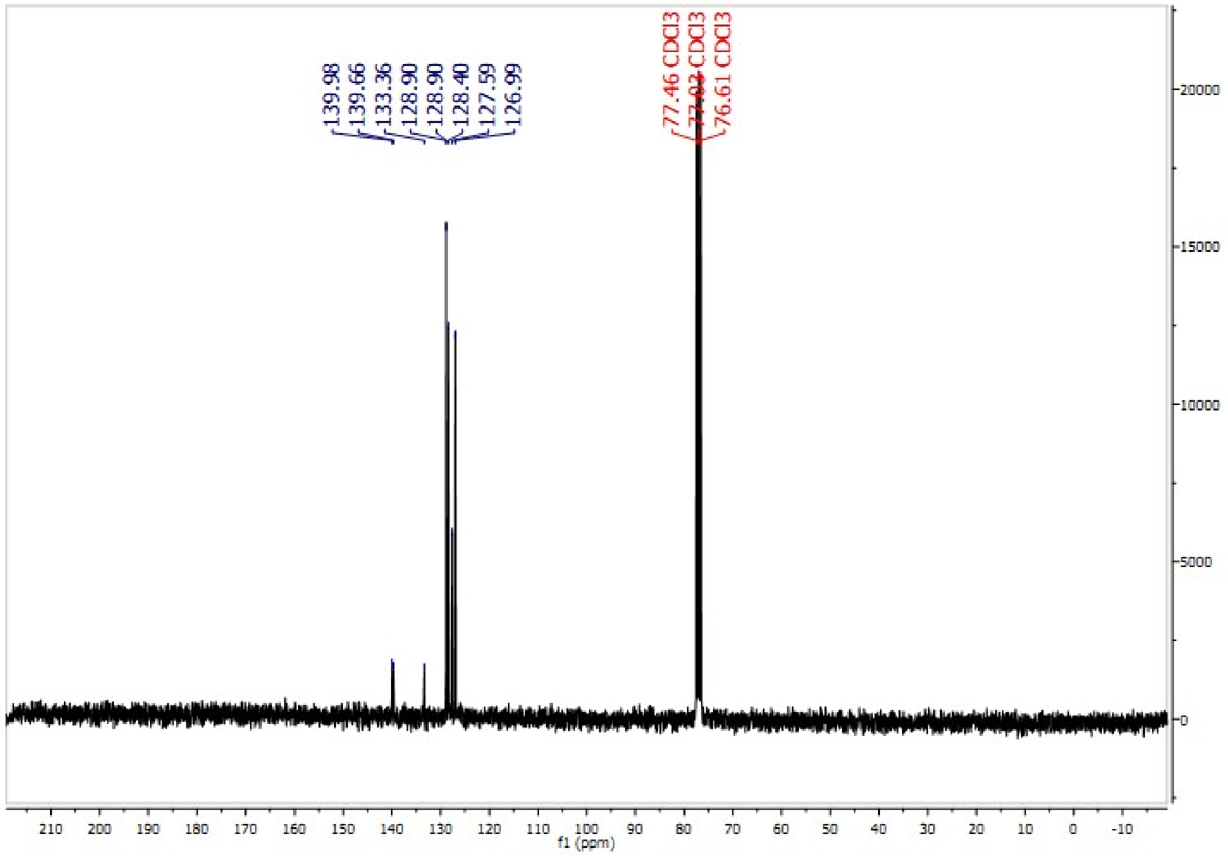

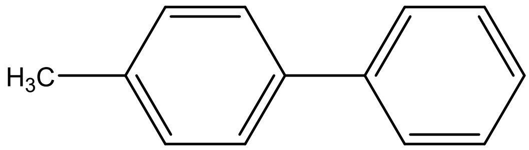

4-methyl-1,1'-biphenyl

^{1}H NMR (300 MHz, δ ppm $CDCl_3$): 7.62 (d, *J*= 9 Hz, 2H), 7.53 (d, *J*= 6 Hz, 2H), 7.46 (t, *J*= 6 Hz, 2H), 7.35 (t, *J*= 6 Hz, 1H), 7.28 (t, *J*= 9 Hz, 2H), 2.43 (s, 3H) ppm.

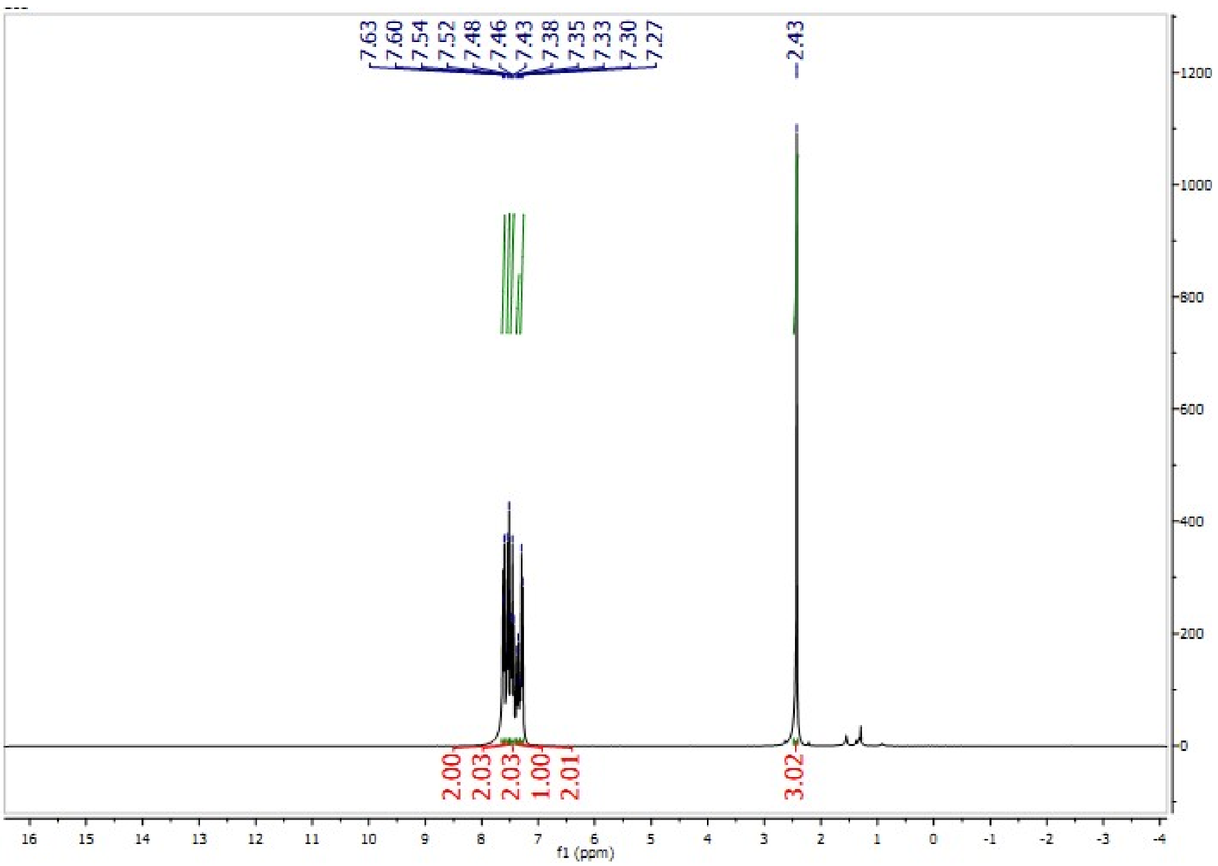

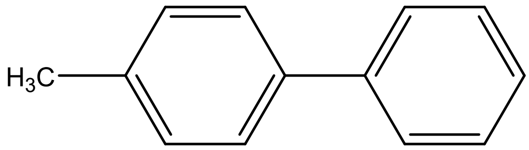

4-methyl-1,1'-biphenyl

^{13}C NMR (100 MHz, $CDCl_3$): 141.2, 138.4, 137.0, 129.5, 128.7, 127.0, 127.0, 21.1 ppm.

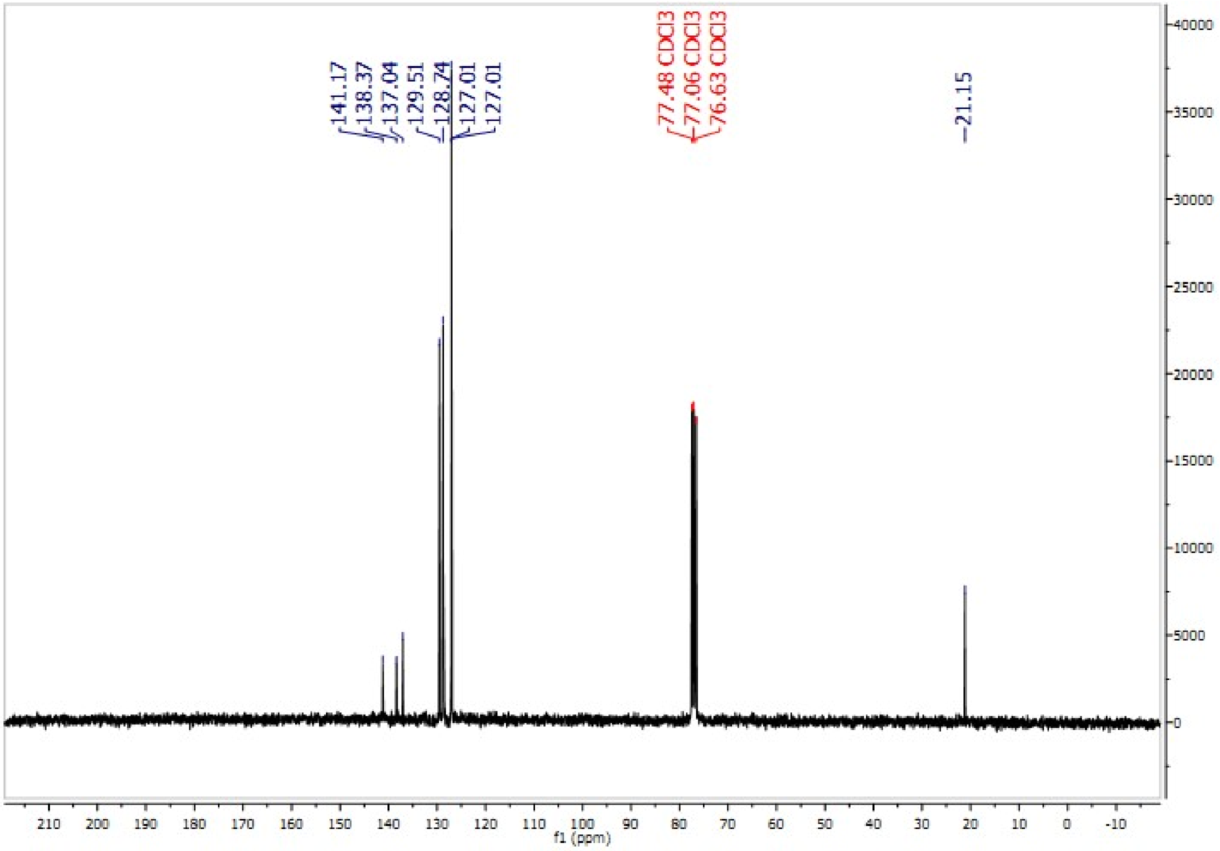

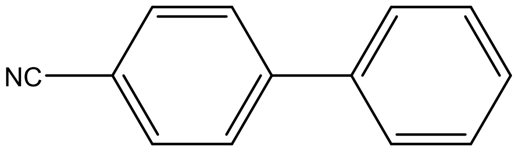

[1,1'-biphenyl]-4-carbonitrile

^{1}H NMR (300 MHz, δ ppm $CDCl_3$): 7.73 (d, *J*= 9 Hz, 2H), 7.68 (d, *J*= 9 Hz, 2H), 7.60 (d, *J*= 9 Hz, 2H), 7.52-7.43 (m, 3H) ppm.

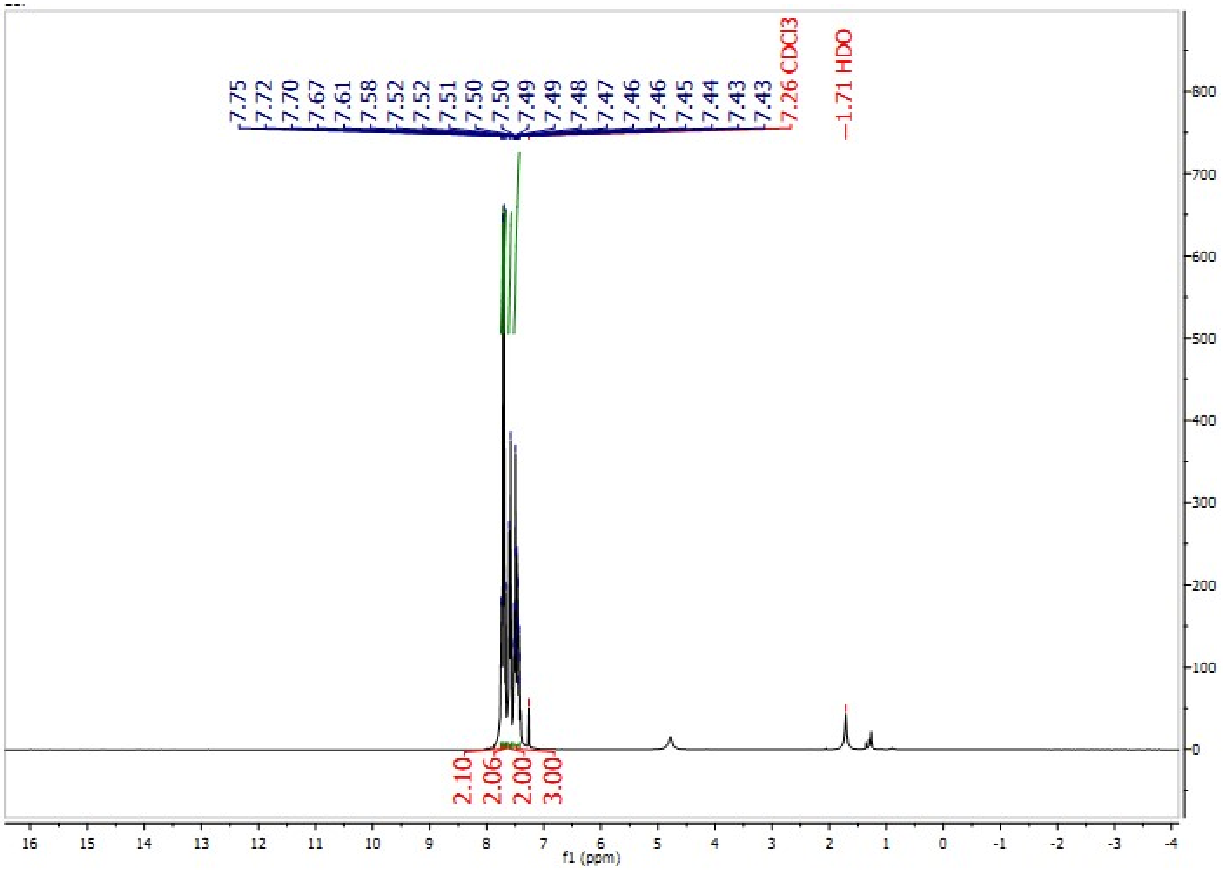

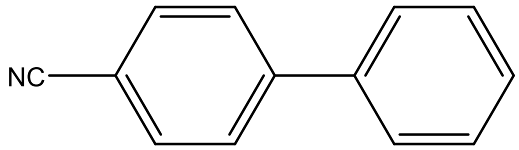

[1,1'-biphenyl]-4-carbonitrile

^{13}C NMR (100 MHz, $CDCl_3$): 145.7, 139.1, 132.6, 129.1, 128.7, 127.7, 127.2, 119.0, 110.9 ppm.

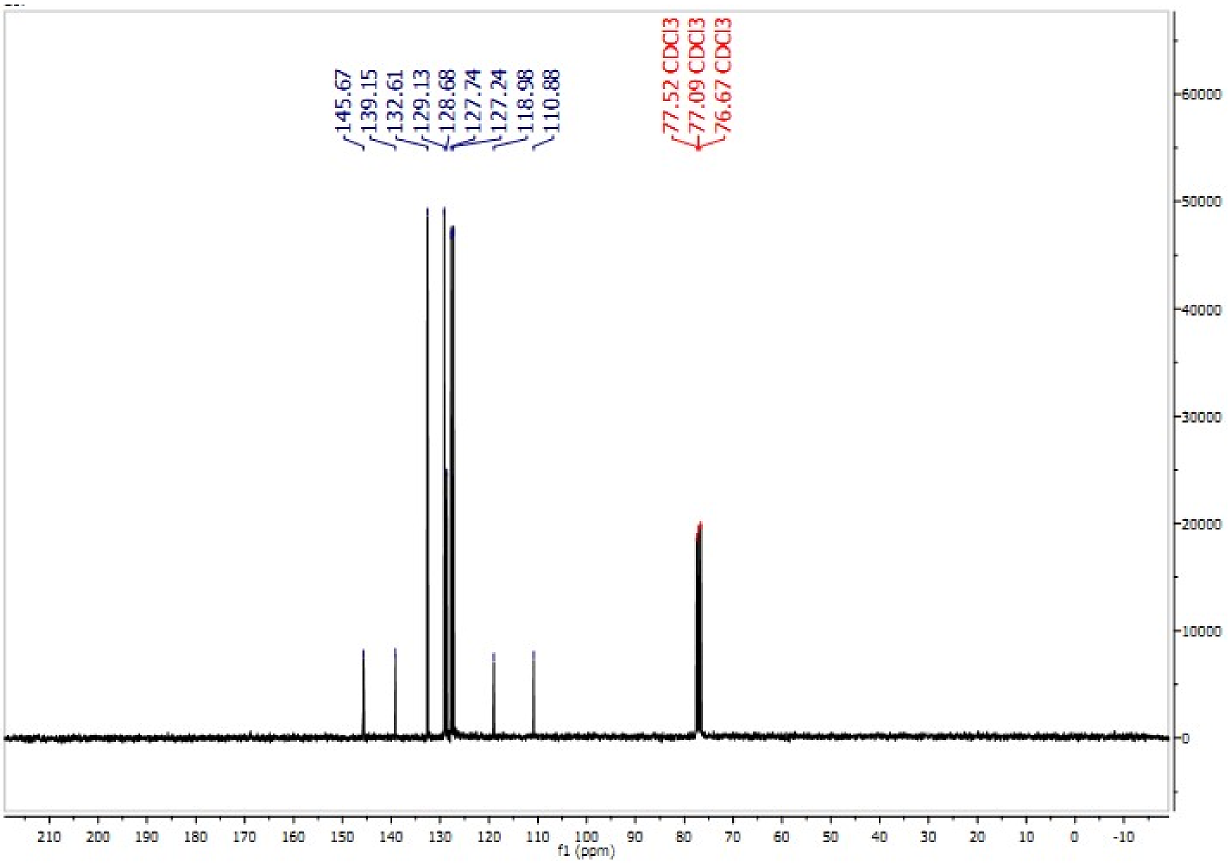

Supplement: RA-016-D5RA08145A-s001 [file RA-016-D5RA08145A-s001.pdf]
